# The transcription of bradyzoite genes in *Toxoplasma gondii* is controlled by autonomous promoter elements

**DOI:** 10.1111/j.1365-2958.2008.06249.x

**Published:** 2008-06

**Authors:** Michael S Behnke, Josh B Radke, Aaron T Smith, William J Sullivan, Michael W White

**Affiliations:** 1 Department of Veterinary Molecular Biology, Montana State University BozemanMT 59717, USA; 2 Department Pharmacology and Toxicology, Indiana University School of MedicineIndianapolis, IN 46202, USA

## Abstract

Experimental evidence suggests that apicomplexan parasites possess bipartite promoters with basal and regulated *cis*-elements similar to other eukaryotes. Using a dual luciferase model adapted for recombinational cloning and use in *Toxoplasma gondii*, we show that genomic regions flanking 16 parasite genes, which encompass examples of constitutive and tachyzoite- and bradyzoite-specific genes, are able to reproduce the appropriate developmental stage expression in a transient luciferase assay. Mapping of *cis*-acting elements in several bradyzoite promoters led to the identification of short sequence spans that are involved in control of bradyzoite gene expression in multiple strains and under different bradyzoite induction conditions. Promoters that regulate the heat shock protein BAG1 and a novel bradyzoite-specific NTPase during bradyzoite development were fine mapped to a 6–8 bp resolution and these minimal *cis*-elements were capable of converting a constitutive promoter to one that is induced by bradyzoite conditions. Gel-shift experiments show that mapped *cis*-elements are bound by parasite protein factors with the appropriate functional sequence specificity. These studies are the first to identify the minimal sequence elements that are required and sufficient for bradyzoite gene expression and to show that bradyzoite promoters are maintained in a ‘poised’ chromatin state throughout the intermediate host life cycle in low passage strains. Together, these data demonstrate that conventional eukaryotic promoter mechanisms work with epigenetic processes to regulate developmental gene expression during tissue cyst formation.

## Introduction

The apicomplexan protozoan *Toxoplasma gondii* is an obligate intracellular parasite capable of invading any nucleated mammalian cell, consequently causing disease in a number of species, including humans. The distribution of parasites across Europe and North America primarily consists of three clonal lineages, Type I, II and III. Although expressed sequence tag (EST) and genomic-based polymorphism studies have shown the genetic diversity between these lineages to be low (Ajzenberg *et al*., 2004; Boyle *et al*., 2006a; Lehmann *et al*., 2006), there are noticeable phenotypic differences. For example, the most virulent strains of the parasite are of the Type I background (Sibley and Boothroyd, 1992). Additionally, differences in migration, growth rate, the ability to differentiate to the cyst forming bradyzoite stage and gene expression have been observed (Radke *et al*., 2006; Taylor *et al*., 2006).

The advent of apicomplexan genome sequencing has led to parallel analyses of the parasite transcriptome with an emphasis on developmental gene expression. There is a solid therapeutic rationale for this focus as clinical sequelae in apicomplexan diseases are often attributable to a specific life cycle stage. In toxoplasmosis, acute pathology is associated with the growth of the tachyzoite stage, while persistent infections are caused by the encysted bradyzoite stage, which is not treatable with current therapies. Global measurements of parasite gene expression demonstrate that primary developmental transitions in the Apicomplexa are accompanied by temporal changes in the level of mRNAs from genes that are dispersed across all parasite chromosomes (Cleary *et al*., 2002; Singh *et al*., 2002; Radke *et al*., 2005). Stage-specific mRNA expression accounts for a large fraction of *Plasmodium* transcripts and there was little shared expression between stages: nearly 200 genes were expressed in the gametocyte, 41 were sporozoite-specific and 20% of all transcripts were specific for the intraerythrocytic cycle (reviewed in Llinas and DeRisi, 2004). Proteomic studies appear to confirm these results as over half of the 948 proteins detected in a recent proteomic analysis were uniquely expressed by a single developmental stage (Hall *et al*., 2005). A global comparison of serial-analysis-of-gene-expression (SAGE) tags (Velculescu *et al*., 1995) from primary Type III strain libraries in *Toxoplasma gondii* demonstrate that unique changes in mRNA levels characterize each developmental transition captured (Radke *et al*., 2005). SAGE tags specific to each developmental stage encompass 23% of the total tags sequenced and ranged from 1.5% to 5.3% of the tags in each library. Transcriptional exclusivity in developmental transitions revealed by SAGE and microarray analysis supports the notion that apicomplexan development follows a hierarchical order that is borne out by temporal changes in gene expression profiles. Altogether, global analysis of gene expression suggests that transcription initiation is a major regulatory mechanism in apicomplexan parasites.

Studies in *Plasmodium* and *Toxoplasma* indicate that the general eukaryotic transcriptional machinery whereby RNA polymerase II transcribes protein encoding genes in association with general transcription factors (GTFs) is relatively conserved and active in these parasites (Li *et al*., 1989; Fox *et al*., 1993; Coulson and Ouzounis, 2003; Coulson *et al*., 2004; Callebaut *et al*., 2005; Meissner and Soldati, 2005; Militello *et al*., 2005). Recently, a combination of two-dimensional hydrophobic cluster analysis and profile-based search methods using PSI-BLAST have extended earlier investigations (Coulson *et al*., 2004; Meissner and Soldati, 2005) such that ∼60% of the known eukaryotic GTFs are now recognized in the *Plasmodium* genome, including important components of the TFIID complex (Callebaut *et al*., 2005). Similar computational surveys demonstrate the presence of GTFs in the *Toxoplasma* genome (Meissner and Soldati, 2005; White *et al*., 2007). Sets of proteins that work in concert with the general transcriptional machinery are the activating transcription factors (ATFs). In higher eukaryotes, ATFs initiate gene expression by binding to *cis*-acting (regulatory) promoter element(s) and recruiting chromatin remodeling complexes (Featherstone, 2002; Li *et al*., 2004), which create a more favourable environment for the assembly of the RNA polymerase complex (White *et al*., 2007). Numerous chromatin remodeling enzymes have been identified in *Toxoplasma* and a variety of histone modifications have been observed at native parasite promoters (Saksouk *et al*., 2005; Smith *et al*., 2005; Gissot *et al*., 2007). By comparison, little is known about apicomplexan ATFs or the mechanisms that activate gene-specific transcription. This is one of the major gaps in our knowledge of apicomplexan biology and filling this deficiency has important implications for understanding parasite development.

In this study, we use low passage *Toxoplasma* isolates to establish in cell culture models that promoters controlling bradyzoite genes have active chromatin configurations in advance of induced changes in mRNA levels. We show that *cis*-acting elements regulating the expression of bradyzoite promoters have definable nucleotide compositions, and these elements retain their activity when introduced into unrelated promoters. We further show that functionally mapped *cis*-elements are bound by protein factors with the appropriate sequence specificity.

## Results

### Global mRNA profiling of Type I, II and III strains

**Fig. 1:**
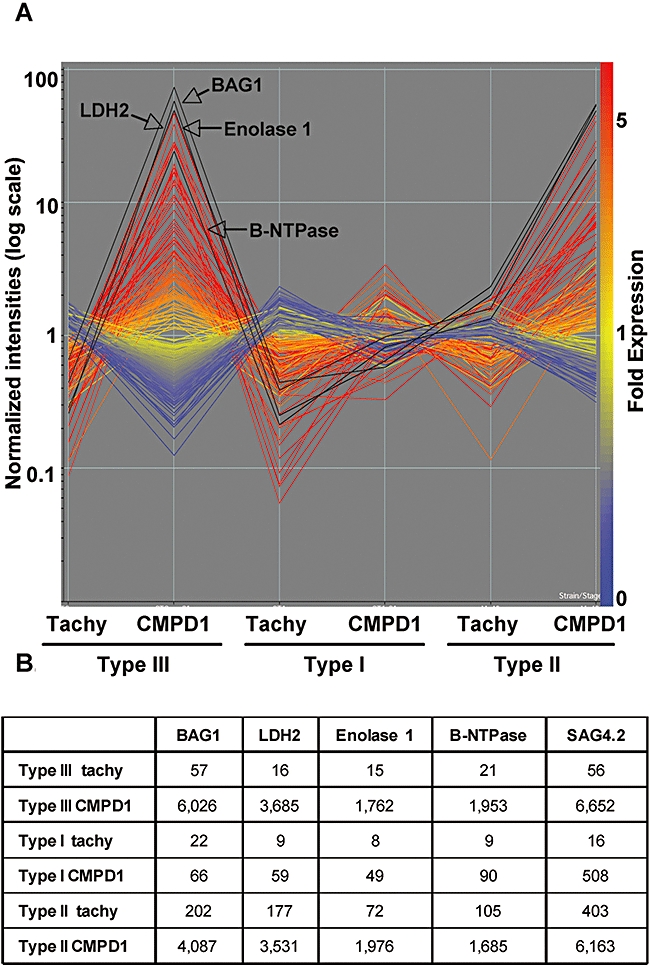
Regulation of mRNA expression during bradyzoite differentiation in low-passage Type III-CTG, Type I-GT1 and Type II-Me49B7 strains. A. The expression of 267 genes that were up- or downregulated by Compound 1 treatment in the Type-II and III strains (3 μM drug for 48 h) are shown (for a list of these genes see Fig. S1). Each line represents one gene in the gene list allowing one to trace the expression of the genes across the experiment. Colouring of the lines (fold scale to the right) is fixed across the graph by the expression level of just one sample, Type III Compound 1 (CMPD1) (yellow-to-red = upregulated; yellow-to-blue = downregulated). Genes induced by Compound 1 include all known bradyzoite genes. BAG1, LDH2, ENO1, SAG4.2 and B-NTPase mRNAs are among the highest expressed mRNAs detected the Type III-CTG and Type II-Me49B7 strains induced by Compound 1 (indicated by labels and with line graphs highlighted in black). Note that the group of bradyzoite genes that are coloured in red or highlighted in black based on the mRNA expression in the Type III-CTG strain are minimally increased in Type I-GT1 parasites exposed to Compound 1. Each data point shown represents RMA-normalized values from two independent biological replicates. B. RMA-normalized fluorescent values for five known bradyzoite genes are listed for three strains maintained as tachyzoites or induced by Compound 1.

Previous studies suggested that specific bradyzoite genes were differentially expressed in the three major lineages common to North America and Europe (Radke *et al*., 2006). In order to determine if these differences extended to the global transcriptome, we characterized whole-cell mRNA levels in both tachyzoite and bradyzoite populations from three primary strain isolates differentiated *in vitro*. It is well understood in the field that long passage history is associated with the loss of developmental competence (Frenkel *et al*., 1976). Therefore, the Type I-GT1, Type II-Me49B7 and Type III-CTG strains studied here were maintained at < 20 cell culture passages from the oocyst stage and are capable of completing both intermediate and definitive host life cycles. These experiments used newly constructed Affymetrix GeneChips that include probe sets for ∼8000 genes, and we assessed the induction of bradyzoite genes using a strong inducer of bradyzoite differentiation, Compound 1 (Radke *et al*., 2006). All known bradyzoite genes (Fig. 1; e.g., BAG1:ToxoDB gene #55.m00009, LDH2:80.m00010, ENO1:59.m03411 and B-NTPase:42.m03416 are indicated) were strongly induced in both Type II-Me49B7 and III-CTG strains, while the Type I-GT1 strain retained a greater tachyzoite character under Compound 1 induction based on mRNA expression (and also shows lower cyst wall formation, Radke *et al*., 2006) with the exception of SAG4.2 (72.m00003) mRNA, which showed induction in Type I-GT1 under these conditions. All genes have been listed in the clustered heat map presented in Fig. S1. It was interesting that basal expression of bradyzoite genes was generally higher in tachyzoites of the Type II-Me49B7 strain, while tachyzoites of the Type I-GT1 and III-CTG strains had much lower mRNA levels for the same bradyzoite genes (Fig. 1B). These data confirm the induction of a novel bradyzoite-specific NTPase (B-NTPase) previously discovered by SAGE in a Type III strain (Radke *et al*., 2005) and further validates that bradyzoite gene expression in Type II strains has a higher spontaneous expression (Radke *et al*., 2005; Boyle *et al*., 2006b). Although similar results were obtained for the above mentioned bradyzoite genes when exposing Type II-Me49B7 and III-CTG strains to alkaline media for a similar time frame, Compound 1 and alkaline media have gene expression profiles that are unique to each condition, suggesting that there is a core set of genes common to bradyzoite differentiation regardless of condition as well as sets of genes which are uniquely regulated by each condition (data not shown).

### Histone acetylation status of bradyzoite promoters in low-passage strains

Recent results correlating histone modifications such as acetylation and methylation with promoter activity has raised the possibility of epigenetic control in developmental gene expression in *Toxoplasma* (Saksouk *et al*., 2005; Sullivan and Hakimi, 2006; Gissot *et al*., 2007). Given the difference in bradyzoite gene expression between the three genotypic lineages (Fig. 1), we explored whether differences in mRNA expression might be explained by strain-specific changes in chromatin. Three bradyzoite promoters (BAG1, LDH2 and B-NTPase) were examined for histone acetylation patterns in low-passage strains. Regulation of these genes during natural and *in vitro* bradyzoite differentiation is well documented in the published literature (Bohne *et al*., 1995; Yang and Parmley, 1995) and is evident in SAGE (Radke *et al*., 2005) and EST data sets (Kissinger *et al*., 2003) and in the microarray analysis shown in Fig. 1.

We performed chromatin immunoprecipitation (ChIP) assays on extracts from all three strains using antibodies specific for acetylated histone H3 or H4 based on previously published protocols (Saksouk *et al*., 2005). Bradyzoite conversion efficiencies for the three strains was assessed independently by FITC-*Dolichos biflorus* lectin fluorescence (Coppin *et al*., 2003). Under the conditions of induction used here, Type II-Me49B7 and Type III-CTG strains convert at > 70%, while Type I-GT1 strain infected cultures had < 30% tissue cysts (data not shown) (Radke *et al*., 2006).

**Fig. 2:**
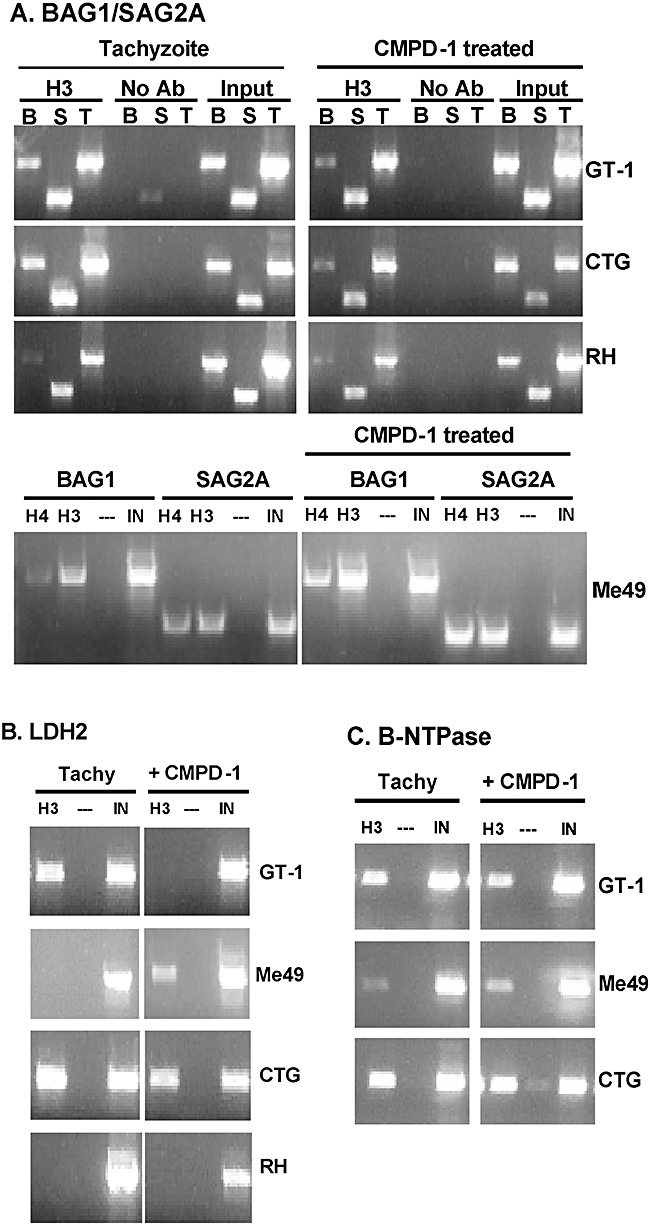
Histone modifications on bradyzoite promoters are poised for activation in low-passage strains. A. ChIP results for the BAG1 (B), SAG2A (S) and β-tubulin (T) promoter regions in three major genetic lineages. The BAG1 promoter was associated with acetylated histones (H3 or H4) in low-passage tachyzoites (left panels) of all major strains (Type I-GT1, Type II-Me49B7 and Type III-CTG) and the levels detected were similar to acetylated H3 levels in the tachyzoite-specific gene SAG2A or the β-tubulin gene, which is constitutively expressed in both tachyzoite and bradyzoites. The levels of H3 or H4 acetylation in these strains was largely sustained in parasites stimulated to differentiate with 3 μM Compound 1 (CMPD1) for 48 h (right panels). Acetylated H3 associated with the BAG1 promoter in the lab-adapted, Type I-RH strain was minimal in tachyzoites or parasites exposed to Compound 1. AcH3, AcH4 = ChIP with antibody specific for acetylated histone H3 or H4; No Ab = no antibody control ChIP; Input = input DNA collected prior to immunoprecipitation. B. ChIP of the LDH2 promoter region in different strains. LDH2 promoter has complex H3 acetylation patterns in different strains. In tachyzoites, we observed strong H3 acetylation associated with the LDH2 promoter in Type I and III strains, while Type II strain tachyzoites the promoter was hypoacetylated (left panels). Following induction by Compound 1, hyperacetylated H3 was detected in the LDH2 promoter from Type II-Me49B7 and III-CTG parasites, while this promoter became hypoacetylated in the Type I-GT1 strain (right panels). In the cell culture adapted Type I-RH strain, the LDH2 promoter was hypoacetylated regardless of stage or culture condition. C. ChIP of the B-NTPase promoter regions in different strains. B-NTPase promoter is associated with substantial H3 acetylation in both tachyzoite and Compound 1-induced extracts in all three low-passage strains with the level of H3 acetylated in Type II-Me49B7 tachyzoites and bradyzoites lower than the Type I-GT1 and Type III-CTG strains.

In low-passage Type I-GT1 and III-CTG strains maintained as tachyzoites (prior to induction), histone H3 acetylation of bradyzoite promoters (BAG1, LDH2, B-NTPase) was extensive and comparable to H3 acetylation of promoter regions flanking the tachyzoite-specific gene, SAG2A (59.m00008), and the constitutive gene, β-tubulin (57.m00003) (Fig. 2A–C), although mRNA levels for these bradyzoite genes are nearly undetectable in tachyzoites of these strains (Fig. 1B). The level of H3 acetylation on these promoters was unchanged or slightly lower following Compound 1 induction in these strains with the LDH2 promoter becoming hypoacetylated in the Type I-GT1 strain. Acetylation of histone H3 was detected on the BAG1 and B-NTPase promoters in Type II-Me49B7 strain tachyzoites (Fig. 2A and C), although these levels were lower and acetylated H3 was not detected associated with the LDH2 promoter in Type II-Me49B7 tachyzoites (Fig. 2B). Upon Compound 1 induction, H3 acetylation was observed on all three promoters in the Type II-Me49B7 strain and acetylated H4 also increased in association with the BAG1 promoter in this strain (Fig. 2A). Interestingly, LDH2 and BAG1 promoters in the Type I-RH strain (Fig. 2A and B) were associated with significantly less H3 acetylation than any of the low-passage strains. H3 acetylation of the BAG1 and LDH2 promoters in Type I-RH was significantly reduced (Fig. 2A) or undetectable, respectively, in the tachyzoites as well as induced bradyzoites (Fig. 2B).

In general, these results are consistent with the pre-activation, or ‘poised’ nature, of bradyzoite promoters in low-passage isolates, while long-term passage in cell culture may lead to histone hypoacetylation on these same promoters. However, the chromatin differences between tachyzoites of the three strains (particularly the lower active chromatin associated with the Type II-Me49B7 strain) was surprising given the clearly higher basal expression of BAG1, B-NTPase and LDH2 mRNAs detected in Type II-Me49B7 tachyzoites (Fig. 1B).

### Adaptation of the dual luciferase reporter assays to the *Toxoplasma* model

The lack of tight correlation between chromatin state and mRNA expression during bradyzoite differentiation in Type II and III strains indicates that other promoter mechanisms are likely key to the changes in developmental gene expression. Transcription studies in *Toxoplasma* are relatively limited with only a few promoters examined. Given this deficiency, we adapted the dual luciferase model (firefly/*Photinus pyralis* and renilla/*Renilla reniformis* luciferase) (Promega, 2006) for use in *Toxoplasma* in order to undertake a detailed mapping study of several bradyzoite promoters. The basic construct for these studies features a variable length promoter fragment that includes the 5′-untranslated region (UTR) as *cis*-elements may lie on either side of the transcription start (Chow and Wirth, 2003). The promoter−5′-UTR fragment is produced by PCR with small recombination sites (attB) incorporated at each end for modular cloning. The fragment is cloned 5′ of the firefly luciferase ORF by recombination (the 3′-attB2 site is translated; Hartley *et al*., 2000). The 3′-UTR and poly(A) sites are contributed by the *Toxoplasma* dihydrofolate reductase-thymidylate synthase (DHFR-TS) gene (Matrajt *et al*., 2004) contained in the vector as our studies, and those of others (Bohne and Roos, 1997; Bohne *et al*., 1997; Kibe *et al*., 2005) demonstrate the 3′-UTR is not required for developmental gene expression. The recombination cloning model (Hartley *et al*., 2000) used here improves flexibility and allows rapid cloning of multiple promoter regions and straightforward deletion and substitution mutagenesis (Carey and Smale, 2000) to identify regions containing bradyzoite gene *cis*-elements.

**Fig. 3:**
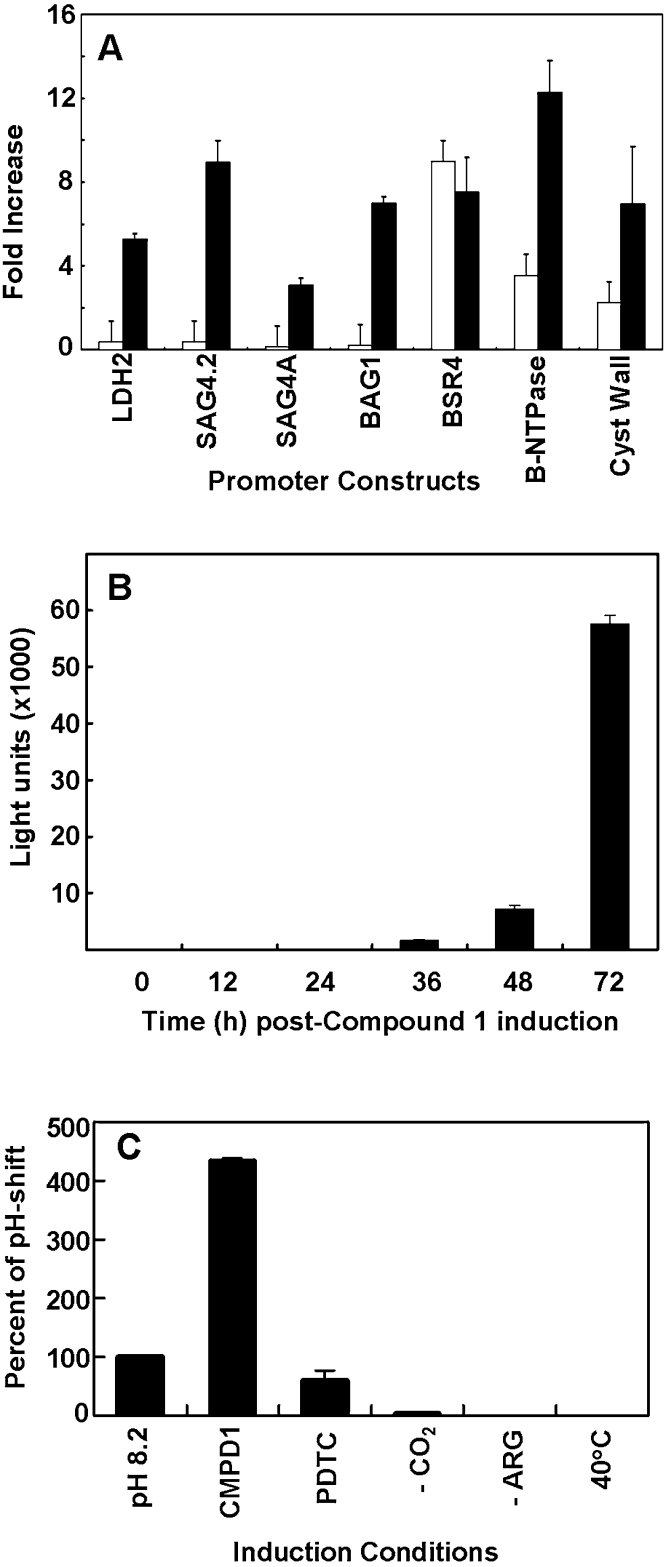
Intergenic genomic regions control developmental gene expression. A. Promoter regions from bradyzoite genes are strongly induced by 3 μM Compound 1 treatment. Several bradyzoite promoters engineered to drive the expression of firefly luciferase were tested under tachyzoite (open bars) and bradyzoite induction (solid bars) conditions. Type III-VEG_msj_ tachyzoites were electroporated with the appropriate plasmid combination (see *Experimental procedures*), inoculated into HFF monolayers (T25cm^2^ flasks), allowed to grow overnight, and then experimental flasks were placed under 3 μM Compound 1 for 48 h. Four readings were collected for firefly and renilla luciferase activity for each promoter construct. Fold increase in luciferase activity in comparison to α-tubulin-renilla controls are plotted for each promoter construct tested under tachyzoite or bradyzoite-induction conditions. All promoters exhibited strong induction following Compound 1 induction with the exception of BSR4, which has been shown to be equally expressed in tachyzoites and bradyzoites (Van *et al*., 2007). B. Integrated promoter constructs in transgenic parasites display natural developmental expression. Clone Type II-Prugniaud-IC2 expresses an integrated copy of the firefly luciferase controlled by the BAG1 promoter (−1197 bp region). Type II-Prugniaud-IC2 parasites were inoculated into HFF monolayers, allowed to grow overnight and then placed under 3 μM Compound 1. Infected monolayers were assessed for luciferase activity (average of four readings) at the indicated time points. Luciferase induction is first observed between 24 and 36 h and increases dramatically in parallel to native BAG1 protein (Radke *et al*., 2006). C. Differentiation conditions vary significantly in their strength of induction. Type II-Prugniaud-IC2 parasites (2 million) were inoculated into HFF monolayers, allowed to grow overnight and then placed under six different induction conditions (see *Experimental procedures*). Drug concentrations: Compound 1 = 3 μM or pyrolidine dithiocarbamate = 100 μM. Infected monolayers were assayed for luciferase activity (4-readings/condition) using standard protocols (Promega manual #TM058) at 72 h post-treatment. Results were calculated as ratios of induced/untreated control (e.g. Compound 1 = 20 970 normalized light units, pH 8.2 = 4838 units, and PDTC = 2900 units) and then graphed in comparison to the pH 8.2 values set to 100%. The order of strength of induction: Compound 1 > pH 8.2 > PDTC > CO_2_ and arginine depletion > high temperature.

To confirm the role of intergenic regions in regulating developmental gene expression in *Toxoplasma*, 16 5′-intergenic flanking regions from previously known bradyzoite- and tachyzoite-specific genes were tested as well as the promoter region flanking B-NTPase (Radke *et al*., 2005). Promoter regions comprising 1–1.5 kbp of 5′-flanking region including the 5′-UTR were fused to the firefly luciferase via recombination cloning and constructs were evaluated in a transient transfection format using the α-tubulin-renilla luciferase (tubulin promoter comprised 2700 bp 5′-flanking region) cotransfection control to normalize differences in electroporation efficiency. Native α-tubulin mRNA levels are relatively unchanged during differentiation (Dzierszinski *et al*., 2001) and varied less than twofold in parasites transiently transfected and induced to enter the bradyzoite pathway with Compound 1 or pH 8.2 media (data not shown). Luciferase expression is induced under Compound 1 conditions for six bradyzoite promoter constructs; LDH2, SAG4.2, SAG4A (72.m00004), BAG1, B-NTPase and cyst wall 65 kDa protein (59.m00006) (Fig. 3A; all primers used for promoter constructs in these and all experiments below are listed in Table S2). Note that the BSR4 (641.m01561) promoter construct was constitutively expressed in these assays, which is consistent with the SAGE frequencies obtained for this gene and the revised assignment of this mRNA as being translationally controlled (Knoll and Boothroyd, 1998; Van *et al*., 2007). A number of other promoters tested had moderate to high levels of expression in tachyzoites (Fig. S2) including SAG1 (p30, 44.m00009), fructose 1,6 bisphosphate aldolase (50.m00005), GRA6 (63.m00002), ROP4 (83.m02145), GAPDH (83.m00003), MIC3 (641.m00002) and IMC1 (44.m00004) as well as DHFR and α-tubulin, which have served as controls for the studies below. Thus, each promoter region tested conveyed expression in the appropriate developmental stage where mRNA levels are highest as reflected in the frequency of SAGE tags (Radke *et al*., 2005) or microarray data in Fig. 1A. These results indicate that *Toxoplasma* 5′-intergenic regions contain regulatory elements that control developmental gene transcription.

To verify that the transient expression of the promoter–luciferase constructs were authentically regulated when integrated into the *Toxoplasma* genome, we have developed several transgenic lines from the Type II-Prugniaud strain (cyst forming) that possesses an integrated copy of the firefly luciferase coding region under control of the BAG1 promoter (results for clone IC2 is shown in Fig. 3B). Induced luciferase protein expression in these strains followed a kinetics (Fig. 3B) that paralleled native BAG1 mRNA and protein expression (Radke *et al*., 2006), and luciferase could be colocalized in parasites induced to express native BAG1 by two colour immunofluorescence (data not shown). Taking advantage of the sensitivity afforded by the luciferase reporter, we evaluated the relative strengths of known methods to induce bradyzoite differentiation (Fig. 3C). Luciferase activity ranged ∼3000-fold in these experiments and parasite growth-inhibition consistently correlated with increased luciferase activity (Bohne *et al*., 1997). The relative strength of induction was: 3 μM Compound 1 (Donald *et al*., 2002; Radke *et al*., 2006), > pH 8.2 media (Weiss *et al*., 1995), > 100 μM pyrrolidine dithiocarbamate (Djurkovic-Djakovic *et al*., 2005), > CO_2_ depletion (Bohne and Roos, 1997), > low-arginine (Fox *et al*., 2004), > high temperature and (Soete *et al*., 1994). These results demonstrated that Compound 1 was comparable to or greater than alkaline media in stimulating luciferase expression in Type II-Prugniaud-IC2 (see also Radke *et al*., 2006). By comparison, we did not observe strong induction of luciferase activity using published methods of arginine (Fox *et al*., 2004) or CO_2_ depletion (Bohne and Roos, 1997), or high temperature as these methods were not as effective under the relatively high moi (1:1) and short time frame we used in these experiments (48 h). We also observed a lack of growth inhibition using Arg and CO_2_ conditions, and high temperature conditions that are congruent with the low levels of luciferase reporter expression detected in these cultures.

### Sequential and internal deletion mapping of bradyzoite promoters

**Fig. 4:**
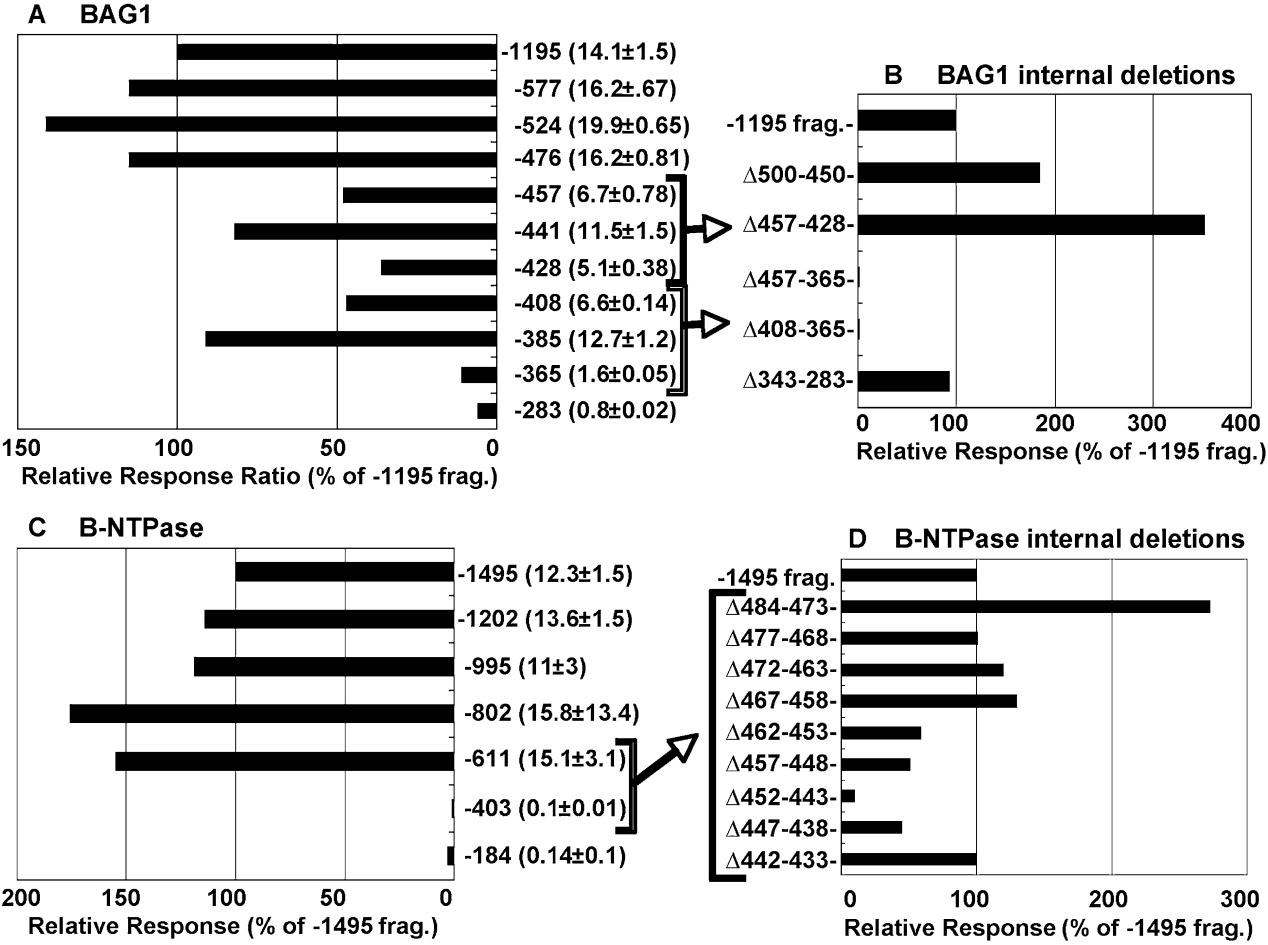
Sequential and internal deletion of the BAG1 and B-NTPase promoters. Transient transfections were performed in duplicate and firefly and renilla luciferase activity was assayed sequentially in each of the samples. Firefly luciferase results were normalized by α-tubulin-renilla levels and graphed as the induced level of expression as compared with the full-length promoter construct (relative response ratio). For each sequential deletion construct, fold change was also determined with respect to the level of luciferase expression in the α-tubulin-renilla control (fold change values and standard deviations in parentheses are listed adjacent each deletion construct). Nucleotide positions in these deletion studies are referenced with respect to the start of translation (+1) in each construct. A. Results of sequential deletion of the BAG1 promoter compared with the full-length 1195 bp promoter construct. B. Results of internal deletion of the BAG1 promoter. Note the regions identified by sequence deletion are referenced by arrow in this internal deletion series. C. Results of sequential deletion of the B-NTPase promoter (fold change values also included) with respect to the 1495 bp full-length promoter. D. A series of overlapping 10 bp internal deletions further refine the required sequence elements first identified by the sequential deletion between −611–403 bp in this promoter (see arrow).

Experimental identification of transcription factors responsible for bradyzoite gene expression follows a logical sequence that begins with careful mapping of the nucleotide elements in promoters using *in vitro* functional assays. To determine whether *cis*-element sequences responsible for the induction of bradyzoite genes in Type II and III strains could be mapped to a high resolution in *Toxoplasma*, we undertook a study to map five different bradyzoite promoters in a Type III strain using the dual-luciferase assay. Sequential deletion of promoters controlling BAG1, LDH2, B-NTPase, SAG4.2 and cyst wall 65 kDa protein (Fig. 4 and Fig. S3) demonstrates that regulatory *cis*-elements have definable locations that are positioned relatively close to the start of transcription in each promoter. Major transcriptional start sites occur at −284 with respect to BAG1 (Bohne *et al*., 1995) and at −330 bp for LDH2 and −370 bp for B-NTPase (mapped by 5′-RACE, data not shown).

Sequential deletion of the BAG1 promoter identified a region between −385 and 365 bp with respect to the BAG1 translational start (Fig. 4A). Further internal deletion mapping confirmed this region as being important to BAG1 promoter induction (Fig. 4B), while deletions on either side of this element had little or no effect on the induction of luciferase expression. It should be noted that the *cis*-elements identified here falls close to a region previously linked to bradyzoite induction in the BAG1 promoter (Bohne *et al*., 1997). Utilizing a similar deletion strategy, we identified the region −611–403 bp as important for induction of the B-NTPase promoter (Fig. 4C). A final series of overlapping 10 bp deletion constructs further resolved the inducible element in the B-NTPase promoter to nucleotides −452–443 bp from the translational start (Fig. 4D).

Sequential deletions in the LDH2 promoter associate the region −508–291 bp with bradyzoite induction (Fig. S3A) and additional internal deletion mapping revealed two distinct elements, between −482–427 bp and −391–381 bp (Fig. S3B). Finally, sequential deletions in the SAG4.2 promoter link bradyzoite induction to the region −616–416 bp (Fig. S3C) and a region −899–688 bp in the cyst wall protein promoter (Fig. S3D). Taken together, deletion constructs confirm and refine the location of *cis*-elements mediating bradyzoite gene expression to the promoter regions −385–365 in BAG1, −452–443 in B-NTPase, −482–427 and −391–381 in LDH2, −616–416 in SAG4.2 and −899–688 in the 65 kDa cyst wall protein promoter.

### Site-directed mutagenesis of the BAG1 and B-NTPase promoter

In order to determine the minimal sequence required to induce expression, the BAG1 and B-NTPase promoters were further fine mapped by site-directed mutagenesis. Mutagenesis was carried out in full-length promoter fragments (1495 bp for B-NTPase and 1195 bp for BAG1) using inverse or overlap extension PCR (Ho *et al*., 1989) with the final PCR fragment recombinationally cloned into the firefly luciferase vector. Two mutagenesis strategies were used with targeted transition of three contiguous bases (A–G or T–C) used for the −385–365 bp region of the BAG1 promoter or a 4 bp AAAA substitution used in the −467–438 bp region of the B-NTPase promoter (Table 1). Transient transfection of site-directed BAG1 promoter mutants in Type III-VEG_msj_ strain identified a single 6 bp segment (TACTGG) that was critical for Compound 1 induction (BAG1 promoter mutant #2 and #3, Table 1). By contrast, alteration of TGTG (B-NTPase mutant #3), CAGC (mutant #6) and GCAC (mutant #7) to AAAA within the −458–439 *cis*-element region of the full-length promoter fragment revealed that each was required for induced expression of the B-NTPase promoter. These results indicate that the B-NTPase promoter has two distinct sequence elements, involving a TGTGTG repeat and the 4 bp sequence CAGC. Interestingly, in both of these promoters fine mapping did not identify a true palindromic sequence as being required for the induction of bradyzoite gene expression. Sequence elements similar to those identified in the BAG1 and B-NTPase promoters can be found in the genomic regions important for bradyzoite induction of the LDH2, SAG4.2 and 65 kDa cyst wall promoters (Fig. S4), although mutations in these sequence elements have not yet been functionally tested using the dual luciferase assay.

**Table 1:** Site-directed mutagenesis of the BAG1 and B-NTPase promoter.

	RRR (%)
A. Mutagenesis of the Brady-NTPase −1495 bp promoter	
WT – TGAC GGTG CGTG TGTG CGTG CAGC GCAC TC	100
Mut1 – TGAC **AAAA** CGTG TGTG CGTG CAGC GCAC TC	47
Mut2 – TGAC GGTG **AAAA** TGTG CGTG CAGC GCAC TC	47
Mut3 – TGAC GGTG CGTG **AAAA** CGTG CAGC GCAC TC	3
Mut4 – TGAC GGTG CGTG TGTG **AAAA** CAGC GCAC TC	51
Mut5 – TGAC GGTG CGTG TGTG CGTG **AAAA** GCAC TC	11
Mut6 – TGAC GGTG CGTG TGTG CGTG CAGC **AAAA** TC	15
B. Mutagenesis of the −1195 bp BAG1 promoter	
WT – CGG TAC TGG CCG CAC GGT TT	100
Mut1 –**TAA** TAC TGG CCG CAC GGT TT	118
Mut2 – CGG **CGT** TGG CCG CAC GGT TT	5
Mut3 – CGG TAC **CAA** CCG CAC GGT TT	8
Mut4 – CGG TAC TGG **TTA** CAC GGT TT	47
Mut5 – CGG TAC TGG CCG **TGT** GGT TT	236
Mut6 – CGG TAC TGG CCG CAC **AAC** TT	120
Mut7 – CGG TAC TGG CCG CAC GGT **CC**	90

RRR = relative response ratio as compared with full-length promoter. Bold nucleotides indicate substitutions.

### Different bradyzoite induction conditions act through common promoter *cis*-elements

**Fig. 5:**
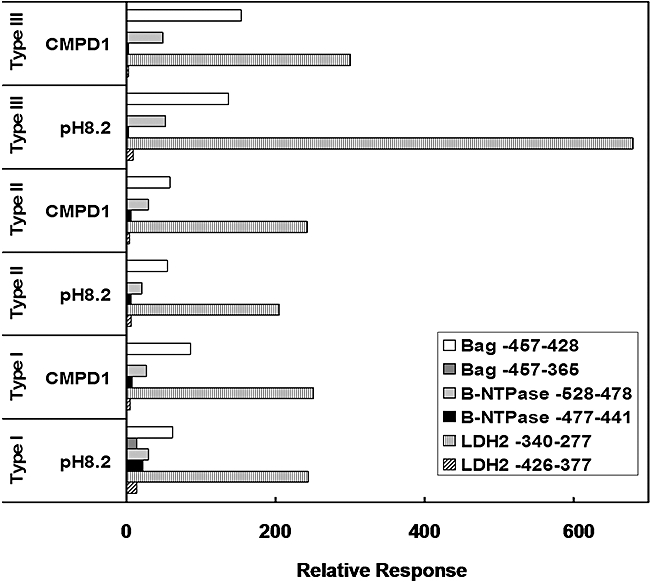
BAG1, B-NTPase and LDH2 promoter induction in Type I, II and III parasites under two different induction conditions. A. Type III-VEG_msj_, Type II-Me49B7 and Type I-RH strains were transiently transfected in duplicate and induced by 3 μM Compound 1 (CMPD1) or pH 8.2 media conditions (48 h). Infected monolayers were harvested at the appropriate times (parasite + HFF cells), lysates prepared and luciferase activity was determined (4-readings/condition). Firefly luciferase results are normalized by α-tubulin-renilla levels and reported as the relative induced expression levels in reference to the corresponding full-length promoter (relative response ratio; fold change values for these experiments are listed in Table S1). Three promoters were tested, BAG1, B-NTPase and LDH2, with two internal deletion constructs compared with three promoter constructs that show maximum inductive activity (BAG1 −1195, B-NTPase −801 and LDH2 −708). Internal deletion in the BAG1 −457–365 bp, B-NTPase −477–441 bp and LDH2 −426–377 bp promoters of the critical *cis*-elements ablate induction of these promoters in all three strains regardless of the induction condition, while neighbouring deletions in each promoter retained at least 50% of the activity of the fully active promoter construct. Each control internal deletion behaved similarly in each strain including the strong induction observed when a putative repressor element that lies between −340 and 277 bp of the LDH2 promoter was deleted.

Characterization of the bradyzoite *cis*-elements in the previous mapping studies used a single induction method and a Type III strain. To determine whether the promoter sequences identified are the key induction elements in these promoters, we compared the two most effective methods for inducing bradyzoite gene expression (Compound 1 versus pH 8.2 media Fig. 3C) in the Type I-RH, II-Me49B7 and III-VEG_msj_ strains. Luciferase activity from the full-length construct for BAG1, B-NTPase and LDH2 promoters was induced to a similar level in Type II and III strains under Compound 1 or alkaline media (Fig. 5 and Table S1). The induction levels of the full-length promoter construct in the Type I-RH strain were on average threefold lower (Table S1) consistent with the reduced efficiency we have observed in bradyzoite switching for Type I strains (Radke *et al*., 2006). Interestingly, there was no detectable difference between strains or induction conditions in transient transfections of key internal deletion constructs (Fig. 5). Transfection of constructs where the key inducible *cis*-element in each promoter had been deleted (BAG1, −457–365 bp; B-NTPase, −477–441 bp; LDH2, −426–377 bp) showed a complete loss of induced expression in all three strains. Control deletions outside these critical *cis*-element regions behaved similarly in Type II-Me49B7 and III-VEG_msj_ strains under the two induction methods. These patterns extended to the apparent repressor element in the LDH2 promoter (−340–277 bp), which when deleted led to a dramatic upregulation of luciferase activity (Fig. 5). These results indicate that the transcriptional mechanisms activated by both induction conditions operate via the same *cis*- regulatory elements in each promoter in all three strains. Diminished expression in Type I-RH is not likely to be due to promoter mutation as Type I-RH promoter constructs were actively expressed when transfected into a Type III strain and there are no polymorphisms in the critical *cis*- element regions for the BAG1, B-NTPase and LDH2 promoters in the three strains (data not shown). Therefore, the explanation for differences in developmental gene expression between the strains is likely to be the response to inductive conditions rather than in the core promoter sequence elements.

### BAG1 and B-NTPase promoter *cis*-elements are sufficient for induced expression

**Fig. 6:**
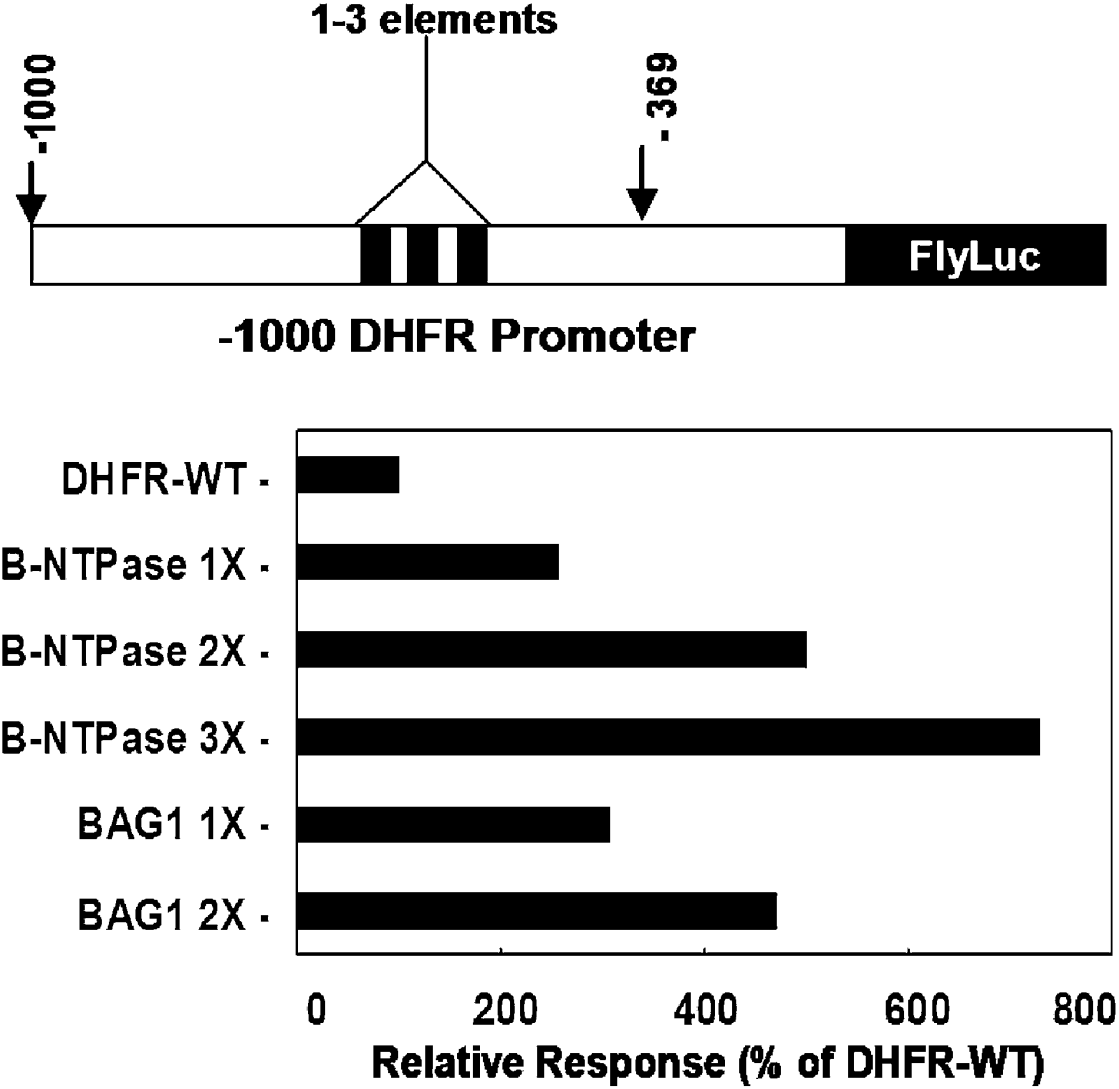
BAG1 and Brady-NTPase *cis*-elements are autonomous in *Toxoplasma*. Copies (1–3×) of the BAG1 or B-NTPase *cis*-element (see Table 1) were introduced into the DHFR promoter (see diagram). The *cis*-elements were placed 100 bp upstream of the major start of transcription at −369 bp (Matrajt *et al*., 2004). The Type III-VEG_msj_ strain was transiently transfected in duplicate and induced by 3 μM Compound 1. Infected monolayers were harvested at the appropriate times (parasite + HFF cells), lysates prepared, and luciferase activity was determined (4-readings/condition). Firefly luciferase results are normalized by α-tubulin-renilla levels and reported as the relative induced expression levels in reference to the corresponding full-length promoter (relative response ratio). No change in luciferase expression was observed under tachyzoite conditions with any construct (data not shown). However, 48 h following 3 μM Compound 1 addition there was a stepwise increase in luciferase activity compared with the WT-DHFR promoter construct based on the number of *cis*-element copies.

We next explored whether the key BAG1 (−386–355) or B-NTPase (−462–438) *cis*-elements were autonomous acting sequences. DHFR-TS is encoded by a constitutive, but low-level transcript that is unaffected by conditions that induce bradyzoite gene expression. In recently conducted microarray studies, DHFR mRNA levels were found to be constitutive in all three clonal strains (Types I-III) during tachyzoite to bradyzoite development and also in growth-synchronized tachyzoite populations (M.S. Behnke and M.W. White, unpublished). We cloned the 1000 bp genomic fragment flanking the DHFR-TS coding region (Matrajt *et al*., 2004) into the luciferase cassette by recombination and then introduced by inverse PCR unique Avr-II/Bgl-II sites 100 bp upstream of the major transcription start site at −369 bp (Matrajt *et al*., 2004). The DHFR-TS promoter with or without the Avr-II/Bgl-II sites was tested for expression in Type III strain tachyzoites and Compound 1-induced parasites and no difference in expression or induction was detected (data not shown). One to three copies of either the BAG1 or B-NTPase *cis*-element were then introduced into the unique Avr-II/Bgl-II sites in the modified DHFR-TS promoter using oligonucleotide linkers. The introduction of either *cis*-element sequence successfully converted the DHFR-TS promoter into one that is induced by Compound 1 (Fig. 6). Following induction with Compound 1 there was a stepwise increase in luciferase activity compared with the wild-type DHFR-TS promoter construct based on the number of *cis*-element copies (Fig. 6). These data indicate the BAG1 and B-NTPase *cis*-elements identified by functional mapping are sufficient to confer increased expression during bradyzoite differentiation.

### Detection of DNA binding activity is specific for B-NTPase or BAG1 functional *cis*-element sequences (EMSA assays)

**Fig. 7:**
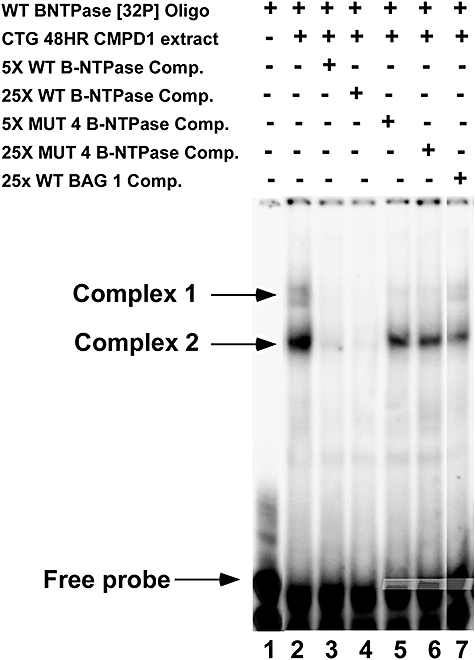
B-NTPase EMSA assay. The B-NTPase *cis*- element forms sequence specific protein–DNA complexes. Incubation of [^32^P]-end-labelled DNA encoding B-NTPase *cis*-element (−467–438 bp, see Table S2 for all primer designs) with nuclear extracts from Type III-CTG parasites induced for 48 h with 3 μM Compound 1 (CMPD1). Note the major complex (complex 2) formed with labelled Brady-NTPase *cis*-element fragment was not diminished by unlabelled B-NTPase mutant #3 sequence (Table 1) or with unlabelled BAG1 *cis*-element. Lane 1, probe alone; lane 2, nuclear extract + probe; lanes 3–7, probe + extract + cold competitor. Lanes 3 and 4, unlabelled wild-type B-NTPase competitor at 5- and 25-fold excess respectively. Lanes 5 and 6, unlabelled B-NTPase mutant #3 (Table 1 and Table S2) at 5- and 25-fold excess. Lane 7, 25-fold excess unlabelled BAG1 *cis*-element.

Eukaryotic gene induction involves transcription factors that recognize specific *cis*- regulatory elements contained within promoters. Using DNA fragments carrying the minimal *cis*- elements functionally mapped in B-NTPase and BAG1 (−467–433 and −385–365 respectively), we performed electromobility shift assays (EMSA) to determine if proteins could be detected that specifically bound these elements. Nuclear extracts prepared from Type III-CTG strain induced to differentiate with Compound 1 were incubated with [^32^P]-labelled DNA fragments and in some cases unlabelled competitor DNA was added to show additional specificity. EMSA assays using a radiolabelled DNA fragment encoding the B-NTPase *cis*-element showed one dominant gel shift complex (Fig. 7, complex 2) with a second minor complex that had a slower mobility (complex 1). Competition with unlabelled wild-type B-NTPase *cis*-element effectively competed the radiolabelled DNA probe (Fig. 7 see 5× and 25× fold competitor, lanes 3, 4), while the addition of unlabelled B-NTPase mutant DNA did not diminish complex formation at two levels of excess competitor (Fig. 7 lanes 5, 6). Consistent with the unique sequences associated with these two *cis*-elements (BAG1 versus B-NTPase, Table 1), 25-fold excess unlabelled BAG1 *cis*-element also did not effectively compete for binding with the labelled B-NTPase probe.

**Fig. 8:**
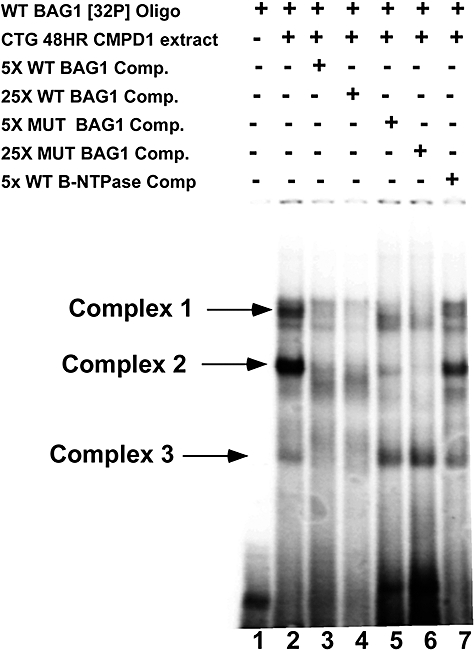
BAG1 EMSA assay. Identification of sequence specific protein–DNA interactions using the BAG1 *cis*- element. Incubation of [^32^P]-labelled BAG1 *cis*-element (−386–355 bp, Table S2 primer designs) with nuclear extracts from Type III-CTG parasites induced for 48 h with Compound 1 (CMPD1). Lane 1, probe alone; lane 2, nuclear extract + probe; lanes 3–7, probe + extract + cold competitor. Lanes 3 and 4, unlabelled wild-type BAG1 competitor at 5- and 25-fold excess respectively. Lanes 5 and 6, unlabelled BAG1 mutant competitor at 5-, 25-fold excess. Lane 7, fivefold excess unlabelled B-NTPase competitor.

The pattern of protein–DNA complexes was more complex when labelled DNA fragment encoding the BAG1 *cis*-element was incubated with Type III-CTG nuclear extracts. Three distinct complexes were observed with this labelled DNA that differed in signal intensity (Fig. 8). To sort out the specificity of these complexes, we evaluated 5× and 25× unlabelled DNA that encoded the wild-type or mutant *cis*-element BAG1 sequence to compete for binding. The result of this experiment showed that a single protein complex appeared to have the appropriate specificity based on functional mapping of the BAG1 promoter (Fig. 8). Unlabelled WT-BAG1 DNA efficiently competed for all protein(s) forming complexes 1–3, whereas mutant DNA competed only for complexes 1 and 2. Proteins forming complex 3 with labelled WT-DNA remained unaffected or were enhanced by the addition of mutant BAG1 DNA, suggesting this is the functionally relevant binding activity. As with the lack of competition of unlabelled BAG1 DNA in the B-NTPase DNA EMSA assays (Fig. 7), we observed little cross-competition with 5× excess B-NTPase DNA when using labelled BAG1 DNA fragments.

## Discussion

There is strong experimental evidence that apicomplexan parasites possess bipartite promoters with basal and regulated *cis*-elements similar to other eukaryotes (Horrocks *et al*., 1998). As a consequence, genomic regions upstream of several *Toxoplasma* genes have served as promoters in vector constructs (White *et al*., 2007). While relatively few of these promoters have been characterized in detail, they appear to work autonomously (without the need for the respective gene specific-3′ regions) via internal sequence elements in a constitutive or stage-specific manner (Soldati and Boothroyd, 1995; Mercier *et al*., 1996; Bohne *et al*., 1997; Roos *et al*., 1997; Nakaar *et al*., 1998; Matrajt *et al*., 2004; Ma *et al*., 2004; Kibe *et al*., 2005). Studies of a few bradyzoite genes are consistent with the principle that mRNA induction is regulated by gene proximal elements in *Toxoplasma*; however, these earlier investigations did not clearly define promoter *cis*-acting sequences that are required and sufficient for the regulation of bradyzoite gene expression (Soldati and Boothroyd, 1995; Mercier *et al*., 1996; Bohne *et al*., 1997; Roos *et al*., 1997; Nakaar *et al*., 1998; Ma *et al*., 2004; Matrajt *et al*., 2004; Kibe *et al*., 2005). Utilizing the dual luciferase assay in *in vitro* induction models, we show that regions no more than 1500 bp upstream of various bradyzoite genes are able to induce luciferase expression in multiple strains and under different bradyzoite induction conditions. *Cis*-acting elements in several bradyzoite promoters were mapped leading to the identification of short sequences that were involved in controlling gene expression. Two of these promoters, BAG1 and B-NTPase were fine mapped to a 6–8 bp resolution (Fig. 4 and Table 1) and these mapped elements were capable of converting a constitutive promoter to one that is induced by bradyzoite conditions. It is interesting to note that these elements do not contain any single nucleotide polymorphism across the three major lineages (data not shown). Protein binding assays demonstrate that these sequences exhibit unique and specific banding patterns, suggesting they are bound by factors, which recognize the particular *cis*- elements (Figs 7 and 8). Altogether these results confirm that transcription initiation is a major mechanism controlling parasite development and these studies are the first to identify the minimal sequence elements that are sufficient for bradyzoite gene expression and to show protein binding specificity for those elements.

The presence of the general eukaryotic transcriptional machinery and a full complement of chromatin remodeling enzymes in apicomplexan parasites are consistent with transcription serving a major role in these parasites. This model is well supported by recent studies that demonstrate primary developmental transitions leading to formation of the *Toxoplasma* tissue cyst are accompanied by a temporally ordered set of transcriptional events from genes that are dispersed across all parasite chromosomes (Cleary *et al*., 2002; Singh *et al*., 2002; Radke *et al*., 2005) (see also Fig. 1). Similar observations have been made in *Plasmodium*, which has lead to the ‘just in time’ hypothesis for those selected genes regulated during development (Bozdech *et al*., 2003; Llinas and DeRisi, 2004). Despite the importance of transcription to apicomplexan life cycles, the mechanistic details of regulated gene expression are poorly understood in these parasites. The abundance of chromatin remodeling machinery conserved in the Apicomplexa has led to the speculation that gene expression might be controlled primarily by epigenetic processes (Saksouk *et al*., 2005; Sullivan and Hakimi, 2006; Hakimi and Deitsch, 2007), but this model lacks elements of specificity that would be needed to explain the strict temporal patterns of gene expression that unfold during parasite development. Data presented here suggest that gene proximal *cis*- elements are required to initiate developmental gene expression, most likely by the binding of gene-specific *trans*-acting factors. Moreover, ChIP assays on the three major lineages indicate that histone acetylation patterns does not always correlate with mRNA expression as there is substantial H3 acetylation associated with a number of bradyzoite promoters in parasites maintained as tachyzoites where mRNA expression is nearly too low to be detected (Kissinger *et al*., 2003; Radke *et al*., 2005) (Figs 1, 2A and B). Acetylation of these bradyzoite promoters is not substantially increased or changed in most of the parasite strains stimulated to differentiate whose conversion to the tissue cyst form is either low (Type I) or high (Type II and III). These results are in contrast to recent reports that suggested a higher correlation between H3 acetylation patterns and changes in developmental mRNA expression in *Toxoplasma* (Saksouk *et al*., 2005; Gissot *et al*., 2007). It is possible that the difference between these studies lies in the early passage strains used here and that long-term adaptation of the tachyzoite stage to cell culture may lead to epigenetic changes in bradyzoite promoters; the hypoacetylated patterns that were prevalent in the classic culture strain Type I-RH shown here are consistent with this idea. These results do not rule out a role for chromatin remodeling in the regulation of bradyzoite gene expression, but rather that changes in histone acetylation alone are not sufficient to account for the transcriptional changes we have measured in primary strains of *Toxoplasma*.

The failure of database mining to find large numbers of transcription factors in apicomplexan genome sequence (Templeton *et al*., 2004; Meissner and Soldati, 2005) has also contributed to the search for alternate explanations of gene regulation in these parasites (Meissner and Soldati, 2005). Given that comparative genomics depends on a level of similarity that might not apply to these parasites, the explanation could lie in the uniqueness of transcription factors in the Apicomplexa (Meissner and Soldati, 2005). This explanation is supported by the recent computationally derived identification of a family of apicomplexan DNA binding proteins containing the AP2-integrase binding domain (Balaji *et al*., 2005; Iyer *et al*., 2008). Alternatively, fewer transcription factors might indicate that only a few factors are required to control the mRNA pool structure unique to each development stage (Radke *et al*., 2005) and that most genes are controlled by a limited but common set of basal transcription factors. One prediction from this model is that transcription in general might be less flexible and this is supported by a nearly complete lack of transcriptional response observed in intracellular *Toxoplasma* tachyzoites that are metabolically altered by drug or gene knockout leading to parasite death (B. Striepen, G.G. van Dooren and M.W. White, unpubl. results). A second prediction of fewer transcriptionally responsive genes is that the pool of mRNAs evaluated by frequency would have a lower complexity. Based on an extensive analysis of the *Toxoplasma* transcriptome by SAGE, we have found a substantially different mRNA pool present in this parasite when compared with higher eukaryotes or to a non-parasitic unicellular eukaryote such as *Saccharomyces* (Radke *et al*., 2005). Relatively few genes (< 5%) account for nearly 75% of all transcripts in the intermediate life cycle stages of this parasite (Radke *et al*., 2005) and the abundant mRNA class in this parasite is populated with transcripts that encode apicomplexa-specific genes. Many of the abundant genes are developmentally regulated and five of these genes were the subject of this study and were shown to be regulated by conventional promoter mechanisms that involve specific *cis*-acting elements.

In conclusion, the lack of knowledge about gene-specific transcription factors is one of the major gaps in *Toxoplasma* biology, and filling this deficiency has important implications for developing treatments that prevent tissue cyst formation. The data presented here increase our understanding of promoter control in the developmental progression of *Toxoplasma* from the tachyzoite to the bradyzoite and sets the foundation needed to utilize biochemical approaches in identifying bradyzoite transcription factors.

## Experimental procedures

### Cell culture and parasite strains

Low passage strains used in these studies were Type I-GT1, the genome reference strain Type II-Me49B7 and Type III-CTG. The following laboratory strains were also used: Type I-RH strain is a well-established lab strain that has lost the ability to complete the two-host life cycle, Type II-Prugniaud strain is capable of robust bradyzoite differentiation and was used to generate stable transgenic lines, and finally, the Type III-VEG_msj_ has a low frequency of spontaneous differentiation when cultured under tachyzoite conditions but is strongly induced to differentiate under a variety of conditions. Type III-VEG_msj_ was used for all deletion/mutagenesis promoter mapping studies. All strains were maintained by serial passage in human foreskin fibroblasts (HFF) cultured in Dulbecco's modified Eagle medium (DMEM, Gibco BRL, Grand Island, NY) supplemented with 1% (v/v) fetal bovine serum (FBS; Atlanta Biologicals, Lawrenceville, CA). Compound 1 was used at a working concentration of 3 μM in 1% FBS DMEM media; pH 8.2 media were made with tricine buffered (45 mM) DMEM without sodium bicarbonate (Cellgro, Herndon, VA) adjusted to pH 8.2 with KOH; l-arginine depleted media were obtained from Specialty Media (Phillipsburg, NJ); parasites shifted to the CO_2_-depleted condition were grown in media made with tricine buffered (45 mM) DMEM without sodium bicarbonate (Cellgro) adjusted to pH 7 with KOH in a CO_2_-free environment; and parasites shifted to high temperature conditions were grown in regular 1% FBS DMEM media at 40°C.

### Microarray analysis

Parasites were purified from host cells as previously described (Roos *et al*., 1994) and RNA was extracted using the Qiagen RNeasy kit: with β-mercaptoethanol and DNase I treatment (Valencia, CA). RNA quality was determined using the Agilent Bioanalyzer 2100 (Santa Clara, CA). A total of 3 μg starting RNA was used to produce cRNA using the Affymetrix One-Cycle Kit (Affymetrix, Santa Clara, CA). Fragmented cRNA (5 μg) was hybridized to the *Toxoplasma gondii* Affymetrix microarray according to standard hybridization protocols (please refer to http://roos-compbio2.bio.upenn.edu/~abahl/Array-Tutorial.html for a detailed description of array design and features). Two hybridizations were done for each sample type. Hybridization data were preprocessed with Robust Multi-array Average (RMA) and normalized using per chip and per gene median polishing and analysed using the software package GeneSpring 7.2 (Agilent Technologies, Santa Clara, CA). The following procedure was used to identify the 267 genes induced by Compound 1: Student's *t*-test grouped by the Compound 1 parameter, variances assumed equal, *P*-value cut-off 0.05, Benjamini and Hochberg False Discovery Rate, using all samples except Type I-GT1 CMPD1 as its lack of induction confounded the use of the Compound 1 parameter) Pearson correlations between independent replicates: strain Type III-CTG tachyzoite = 0.976, strain Type III-CTG-CMPD1 induced = 0.976, strain Type I-GT1 tachyzoite = 0.945, strain Type I-GT1-CMPD1 induced = 0.982, strain Type II-Me49B7 tachyzoite = 0.968, and strain Type II-Me49B7-CMPD1 induced = 0.95. Percentage of expressed probes out of 8131 total probes: Type III-CTG tachyzoite = 68%, Type III-CTG-CMPD1 = 70%, Type I-GT1 tachyzoite = 62%, Type I-GT1-CMPD1 = 75%, Type II-Me49B7 tachyzoite = 70%, and Type II-Me49B7-CMPD1 = 71%.

### Chromatin immunoprecipitation

Freshly lysed tachyzoites or *in vitro* bradyzoites were treated for 10 min with 1% formaldehyde at 37°C, spun and re-suspended in cold lysis buffer (1% SDS, 10 mM EDTA, 50 mM Tris-HCL, pH 8.1, plus protease inhibitors). Samples for ChIP consisted of ∼5 × 10^7^ parasites that were subjected to sonication on ice three times, 10 s each. The following steps were carried out at 4°C unless indicated otherwise. Samples were centrifuged at 13 000 r.p.m. for 10 min, and a portion of the supernatant was saved for input DNA. The rest was diluted 10-fold in ChIP dilution buffer (0.01% SDS, 1.1% Triton X-100, 1.2 mM EDTA, 16.7 mM Tris-HCl, 167 mM NaCl, pH 8.1) plus protease inhibitor cocktail (Sigma P8340). Lysate was precleared with 80 μl salmon sperm DNA protein A agarose for 30 min with agitation. Following centrifugation at 1000 r.p.m. for 1 min, anti-AcH3 or anti-AcH4 (Upstate Biotechnology 06–599 and 06–866 respectively) was added for an overnight incubation. Then 60 μl of salmon sperm DNA/protein-A agarose was added and incubated for 1 h to collect the antibody–protein–DNA complex. The resulting complex was washed 2× with 1.0 ml of each of the following buffers for 5 min: low salt wash buffer (0.1% SDS, 1% Triton X-100, 2 mM EDTA, 20 mM Tris-HCl, 150 mM NaCl, pH 8.1); high salt wash buffer (0.1% SDS, 1% Triton X-100, 2 mM EDTA, 20 mM Tris-HCl, 500 mM NaCl, pH 8.1); LiCl wash buffer (0.25 M LiCl, 1% NP40, 1% deoxycholate, 1 mM EDTA, 10 mM Tris-HCl, pH 8.1). Samples were washed 4× in TE prior to elution in 250 μl elution buffer (1% SDS, 0.1 M NaHCO_3_) for 15 min at room temperature with agitation. After a spin at 1000 r.p.m. for 1 min, a second elution step was performed on the supernatant. NaCl (20 μl of 5 M) was added to the combined eluates and cross-links were reversed by heating for 4 h at 65°C. Samples were then treated with proteinase K and DNA recovered by phenol/chloroform extraction followed by ethanol precipitation. Standard PCRs were carried out with designated primers (see Table S2) using Taq polymerase (Invitrogen), and products visualized on a 1% agarose gel containing 0.5 μg ml^−1^ ethidium bromide. Regions amplified from putative start ATG: BAG1 = −979 to −263, LDH2 = −810 to −43, SAG2A = −438 to −1, B-NTPase = −1280 to −753.

### Promoter deletion, substitution and mutagenesis constructs

In order to construct a renilla version of the ΔCAT-pluc-firefly luciferase (Matrajt *et al*., 2002), the renilla coding region was amplified by PCR with primers that incorporated Avr-II/Pst-1 restriction sites. This DNA fragment was then used to replace the firefly coding region in the ΔCAT-pluc-firefly luciferase plasmid. To prepare destination vectors for cloning various promoter−5′-UTR genomic fragments, we replaced the existing promoter and ΔCAT regions (Kpn1/AvrII) in the ΔCAT-pluc-firefly luciferase and ΔCAT-pluc-renilla-luciferase plasmids with an oligonucleotide linker that contained KpnI/SmaI/AvrII sites. The unique SmaI site was then used to clone the cassette B fragment that carries the ccdB and chloramphenical resistance markers for eventual recombinational cloning (Invitrogen, Carlsbad, CA). The placement of the cassette B in these plasmids enabled recombinational cloning of promoter fragments 5′ of the firefly or renilla coding regions. Promoter fragments included 5′-flanking genomic sequence including the 5′-UTR and the translational start. The attB2 sequence is translated and in frame with the luciferase coding region. The resulting plasmids were renamed DEST-p-firefly parent or DEST-p-renilla-parent and carried the ampicillin resistance marker for selection in bacteria, although no *Toxoplasma* selection marker was included.

Sequential deletion constructs were made by amplifying the appropriate promoter and 5′-UTR regions from Type II genomic DNA for each gene tested using primers containing flanking the attB1 and attB2 sites. The promoter fragment was first cloned into pDONR221 (Invitrogen) via the BP reaction, and after restriction enzyme verification, the promoter fragment was moved into either of the destination vectors above via the LR reaction (Invitrogen). Nucleotide positions in these deletion studies are referenced with respect to the start of translation (+1).

Substitution and site-directed mutagenesis constructs were obtained by alterations of the appropriate pDONR221 entry vector containing the promoter fragment of interest by using this plasmid as a template for inverse PCR where the plasmid was first amplified and then re-ligated following digestion of ArvII sites incorporated into the primer designs. Constructs were verified with restriction enzyme mapping, and then modified promoter moved to the Dest-p-firefly vector via the LR reaction.

### Dual luciferase assay

Parasites were electroporated as previously described (Striepen *et al*., 2002) with a combination of the Dest-p-firefly promoter construct of choice (40 μg) and the control Dest-p-renilla-α-tubulin plasmid (20 μg). Electroporations were performed in duplicate with each electroporated sample split equally into two T25cm^2^ flasks (control and experimental) and allowed to grow ∼16 h at 37°C. Following the period of overnight recovery, bradyzoite inductive media or culture condition was applied to two experimental flasks. Samples were processed for luciferase activity by scraping the infected monolayer, harvesting the cells by centrifugation and re-suspending the cell pellet in 1× Lysis Buffer (Promega, Madison, WI). The lysates were vortexed, incubated at room temperature for 15 min, then centrifuged for 5 min at 14 000 r.p.m. (tabletop microcentrifuge) and kept on ice until assayed for luciferase activity. Firefly and renilla luciferase activity was determined in the same extract using Promega Dual Luciferase Assay reagents according to manufacturer protocols (Promega) and light readings taken on a Lumat LB 9507 luminometer. Four readings were collected for firefly and renilla luciferase activity for each promoter construct. Firefly luciferase results were normalized by α-tubulin-renilla levels and referenced to the induced expression levels of a control promoter construct (relative response ratio) or compared with the control α-tubulin-renilla promoter construct (fold change). All constructs (control, deletion or mutant) were expressed > 10-fold (typically > 100-fold) over non-specific background (30 light units on average) following induction.

### Preparation of BAG1-LUC transgenic parasites

Type II-Prugniaud, was co-electroporated with a plasmid (45 μg) carrying the firefly luciferase region under control of the full-length BAG1 promoter (1195 bp 5′-flanking region) and the plasmid pC3 (5 μg), which encodes the pyrimethamine resistance marker (Donald and Roos, 1993). Following an overnight recovery in normal culture media, the parasites were selected in media containing 1 μM pyrimethamine. Pyrimethamine-resistant parasites were then cloned by limited dilution and clones tested for luciferase activity under Compound 1 or alkaline media induction. Clone IC2 was one of several clones that displayed strong luciferase induction under bradyzoite induction conditions (data not shown).

### Protein extracts and EMSA assays

Type III-CTG parasites induced by 3 μM Compound 1 for 48 h were harvested and protein extracts produced using NE-PER nuclear and cytoplasmic extraction reagents according to manufacturer protocols (Pierce, Rockford, IL). Oligonucleotide probes were hybridized and then end-labelled by incorporation of [^32^P] using 5 units of T4 polynucleotide kinase (USB, Cleveland, OH) and 10 mCi ml^−1^[γ-^32^P]-ATP substrate (Amersham Biosciences, Piscataway, NJ). Unincorporated nucleotides were removed with NucAway spin columns (Ambion, Austin, TX) and the probes stored at −20°C.

EMSA binding reactions contained 200 ng polydI:dC, 12.5 μg BSA, 10 mM DTT, 7.5 mM MgCl_2,_ 67% Buffer D (20 mM HEPES, 20% v/v glycerol, 0.2 mM EDTA, 100 mM KCL, the following were added fresh 0.5 mM PMSF, and 1 mM DTT). Approximately 1 fmol of purified radiolabelled oligonucleotide and 10 μg of protein extract in reaction buffer were incubated on ice for 1 h, loaded directly onto a 4.5% native acrylamide gels (pre-electrophoresed at 20 mA for 1.5 h at 4°C), and the complexes were resolved at 40 mA for 2.5 h at 4°C in running buffer (0.5× TBE, 1% glycerol). The gels were transferred to whatman, dried and exposed to a phosphorimaging screen. All EMSA assays were performed at < 50% probe shift in order to compare the levels of complex formation between reactions.

## Supplementary material

Figure S1 Dose-dependent response of peripheral blood Vδ2^+^ T cells to pamidronate stimulation. This material is available as part of the online article from: http://www.blackwell-synergy.com/doi/abs/10.1111/j.1365-2958.2008.06249.x (This link will take you to the article abstract). Please note: Blackwell Publishing is not responsible for the content or functionality of any supplementary materials supplied by the authors. Any queries (other than missing material) should be directed to the corresponding author for the article.
