# Supplementary material for: The transcription of bradyzoite genes in Toxoplasma gondii is controlled by autonomous promoter elements
Source: Mol Microbiol. 2008 Jun;68(6):1502–18. doi: 10.1111/j.1365-2958.2008.06249.x (PMC2440561; doi:10.1111/j.1365-2958.2008.06249.x)
Supplement: Figure S1 — Dose-dependent response of peripheral blood Vδ2+ T cells to pamidronate stimulation. [file mmi0068-1502-SD1.pdf]

Figure S1. Heat map of 267 genes regulated by Compound 1 treatment. Behnke *et al.*

Genes were cluster in a continuous heat map that has been split into three column groups: the end of column group 1 continues at the top of column group 2 and the end of column group 2 continues at the top of column group 3. The columns within column groups represent the six samples: **1** – Type-III tachyzoite, **2** – Type-III Compound 1 48 hrs, **3** – Type-I tachyzoite, **4** – Type-I Compound 1 48 hr, **5** – Type-II tachyzoite, **6** – Type-II Compound 1 48hrs and are colored by the colorbar at far right (yellow-to-red=up-regulated; yellow-to-blue=down-regulated) .

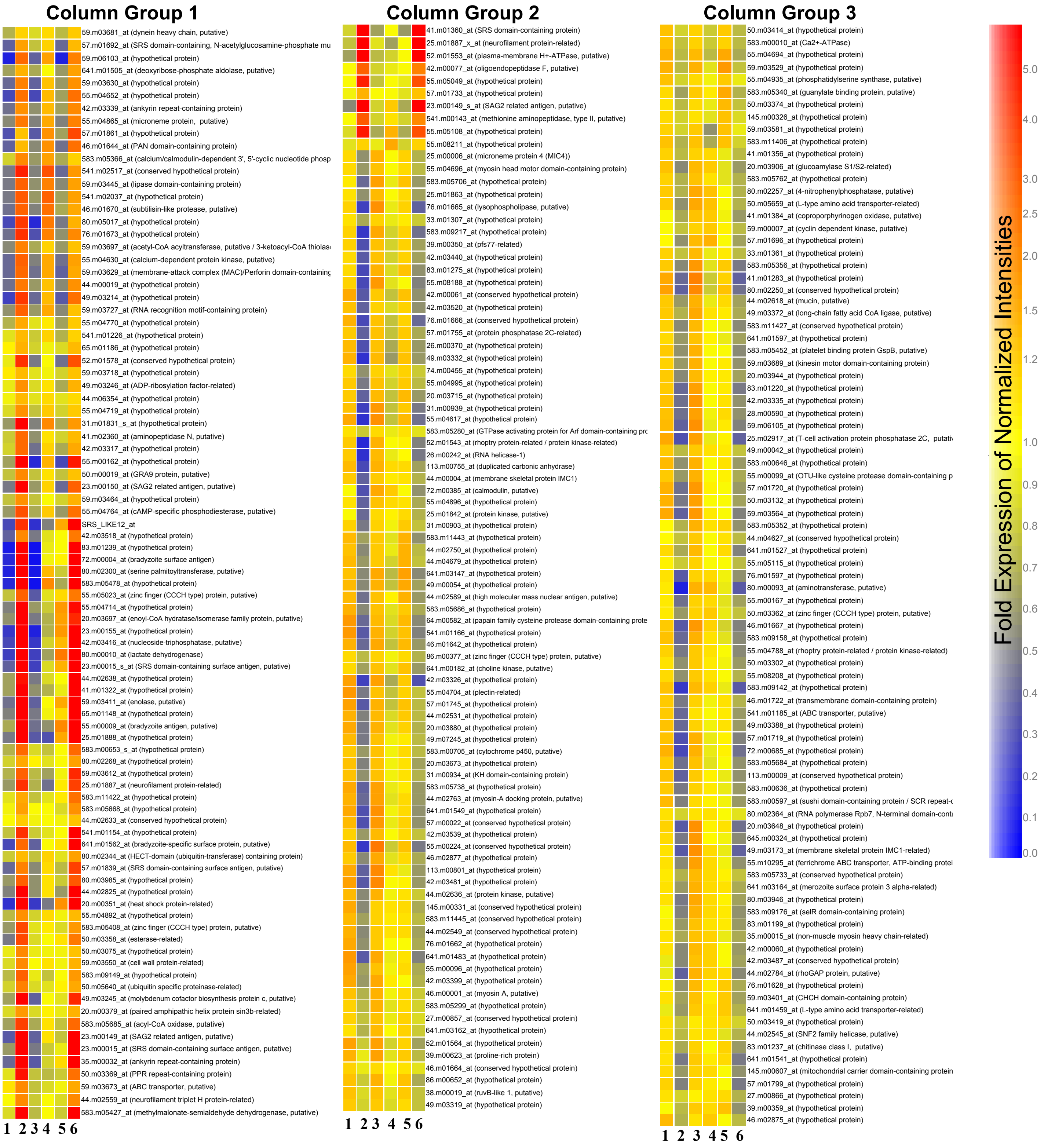

Figure S2. A number of promoters have moderate to high levels of expression in tachyzoites.
Promoter regions (1-1.5 kbp) from various genes were tested for expression in Type III-VEGmsj tachyzoites using the dual luciferase assay. A mixture of 40 μg of the appropriate firefly promoter construct and 20 μg of the control α-tubulin promoter construct was transfected in duplicate and luciferase activity expression measured following 36 h of parasite growth. Light emission values for each promoter tested in the context of driving the firefly luciferase (open bars) and values for the co-transfected control α-tubulin promoter in each experiment (renilla luciferase, solid bars) are plotted on a log scale. Note that while firefly luciferase expression in tachyzoites transfected with the experimental promoters varies over two logs, the expression of the α-tubulin-renilla control construct is reasonably consistent between experiments.

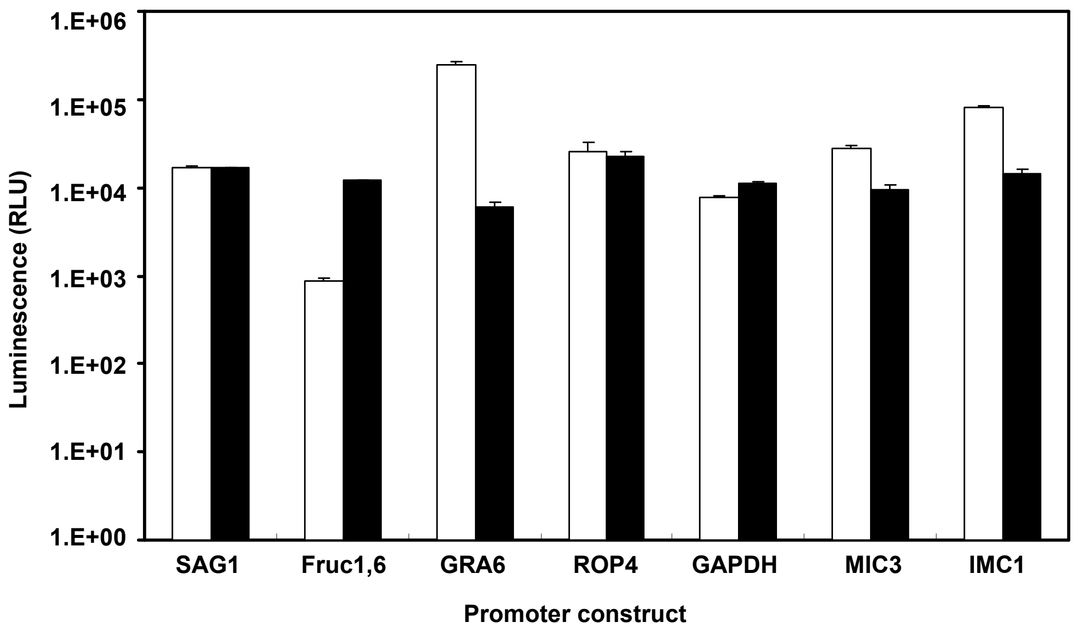

Figure S3. Mapping of the LHD2, SAG4.2, B-NTPase and Cyst-Wall protein promoters. Transient transfections were performed in duplicate and firefly and renilla luciferase activity was assayed sequentially in each of the samples. Firefly luciferase results were normalized by α-tubulin-renilla levels and graphed as the induced level of expression of the indicated full length promoter construct (relative-response-ratio, RRR). For sequential deletion constructs, fold change was also determined with respect to the level of luciferase expression in untreated controls; fold change values and standard deviations in parentheses are included for each sequential deletion construct. Nucleotide positions in these deletion studies are referenced with respect to the start of translation (+1) in each construct. **(A).** Results of sequential deletion of the LDH2 promoter compared to the full length -1509 bp promoter construct. **(B).** Results of internal deletion of the LDH2 promoter with respect to the -708 bp promoter (which is fully active and comparable to the -1509 bp construct). Note the region identified by sequential deletion are referenced by arrow in the internal deletion series. Two discrete sequence spans were observed to be required for induction of the LDH2 promoter. **(C).** Results of sequential deletion of the SAG4.2 promoter (fold change values also included) with respect to the 1,609 bp full length promoter. The loss of promoter activity associated with the sequential deletion between -616-416 caused a loss of relative promoter activity, although the fold change values were less affected. Further internal deletions are necessary to resolve whether this *cis*-element region is a basal rather than inductive element. **(D).** Results of sequential deletion of the cyst wall (65 kDa protein) promoter identified a region between -899 and -688 bp as required for bradyzoite induction. Note also that the deletion between -1099 and -899 leads to a substantial increase in luciferase over the -1501 full length promoter control indicating the presence of a possible repressor element.

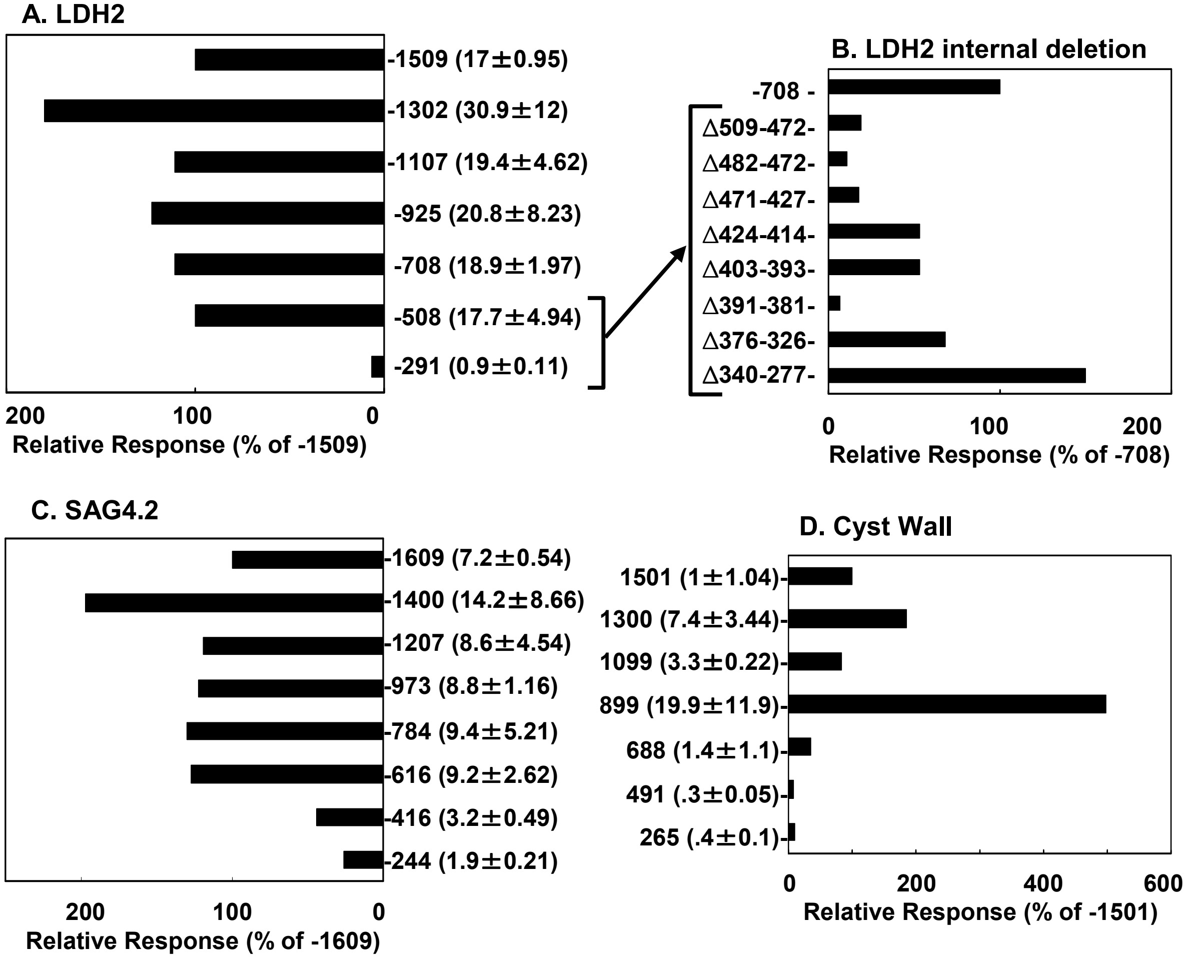

Figure S4. BAG1 and B-NTPase *cis* element sequences can be identified in functionally mapped regions of other bradyzoite promoters.
DNA sequences similar or identical to the minimal *cis* elements required for bradyzoite induction of the BAG1 and B-NTPase promoters can be found in the genomic regions important for bradyzoite induction of the LDH2, SAG4.2, and 65 kDa cyst wall protein promoters.

NTPase cis-elements = TGTGTG CAGC

BAG1 cis-element = TACTGG

SAG4.2 -616-416<br>GGTCGTACAATGTTGTGTACGGTAAAAGCAACAACTGAAAAGTTGTTCCT<br>TGAGCGTTCTCGCTGTCGCCACCGTTGCAGCTGGAATCGGTACCGCGGTC<br>TTCTGATCAGGCACAAAGGCATGATCCCACGATCAGGCAGGCGGCAACAT<br>CCGCAGACATCTACCGCTGGCATACACCGAACTACCACGCAGAAGAAGATG
LDH2 -426-377<br>GGGAGGTACTGCCCGGAGCATGCGTCCGGTTCCAGAACCAGCGCCGGCCG
Cyst Wall -889-686<br>GGAACGACGCTCATCAGCAATCCCGCTTGTCGCATACGCCGTGAATCCGC<br>GTGTCGCATACGATCACCAGTACTAGCAGCCTCGAGGTTTTCGGTGGAGT<br>TGCAGTTGCGTATGCCAGTGCGTCACAGCGCTGAACTACTGGCAGGTAGA<br>CACATCTGGCAAGCTACTGCTGTGTCTGAAGCGTGCCGATGTGTGCGCGTACGC

Table S1. BAG1, B-NTPase and LDH2 promoter induction in Type I, II and III parasites under two different induction conditions (fold change and standard deviation values).
For each construct, fold change was also determined with respect to the level of luciferase expression in the co-transfected α-tubulin-renilla control. Note, the fold change for all three promoter is significantly lower in the Type-I strain as compared to Type-II and III.

	**Type III C1**	**Type III pH8.2**	**Type II C1**	**Type II pH8.2**	**Type I C1**	**Type I pH8.2**
**BAG -1195**	9.28 ± 1.4	9.29 ± 0.52	7.82 ± 0.31	11.92 ± 0.42	2.03 ± 0.03	0.02 ± 0
**BAG -457-428**	14.33 ± 1.73	12.79 ± 0.19	4.5 ± 0.62	6.54 ± 0.07	1.76 ± 0.05	0.01 ± 0
**BAG -457-365**	0.04 ± 0	0.05 ± 0	0.02 ± 0	0.06 ± 0.01	0.02 ± 0	0.003 ± 0
**B-NTPase -801**	6.60 ± 0.14	6.27 ± 2.41	4.20 ± 0.24	4.62 ± 0.24	1.41 ± 0.08	0.04 ± 0
**B-NTPase 528-478**	3.18 ± 0.2	2.94 ± 0.18	1.42 ± 0.25	1.06 ± 0.37	0.37 ± 0.01	0.01 ± 0
**B-NTPase 477-441**	0.17 ± 0.02	0.11 ± 0.03	0.28 ± 0.03	0.29 ± 0.02	0.11 ± 0	0.008 ± 0
**LDH2 -708**	3.35 ± 0.07	0.75 ± 0.03	5.41± 0.31	5.18 ± 0.39	1.00 ± 0.21	0.02 ± 0
**LDH2 340-277**	10.31 ± 1.11	5.10 ± 0.27	13.06 ± 0.39	10.62 ± 0.22	2.51 ± 0.25	0.06 ± 0
**LDH2 426-372**	0.08 ± 0.01	0.06 ± 0.01	0.19 ± 0.02	0.29 ± 0.04	0.05 ± 0	0.003 ± 0

Table S2. Primers used (unique to each figure).

Figure 2 Primers	
Beta-Tubulin Forward	5'-AAAGGTGAGAGGAGGAGTGCGCCTGGATCG-3'
Beta-Tubulin Reverse	5'-TCCTAACACCAACGCACGGGAGCGTTCTGC-3'
BAG1 forward	5'-TCACCATCCAGCTTTCCATTCACTTAACCG-3'
BAG1 Reverse	5'-ATCTGAGAAAACGCTCCGGCCTTCTGCC-3'
SAG2A forward	5'-CGTAAAGCGCGCAGATGACAG-3'
SAG2A reverse	5'-GTTCCGACAAGACAAAGATTG-3'
LDH2 forward	5'-GATCAAACACGGATCTACACCCCTGTTGCG-3'
LDH2 reverse	5'-TAAGTATGTTGCTACGAAAAGCCACCG-3'
B-NTPase forward	5'-TTCAAATAGTGCCGGTCGAGAAGAATTCG-3'
B-NTPase reverse	5'-AAGTAATCCCACTGGGGTGAGTCTAGC-3'

Figure 3 Primers	
alpha-tubulin attB2	5'-GGGGACCACTTTGTACAAGAAAGCTGGGTCCGCCATAAAAGGGAATTCAAGA-3'
alpha-tubulin attB1	5'-GGGGACAAGTTTGTACAAAAAAGCAGGCTTCCAGCATCATCTCTGGAAGCA-3'
LDH2 attB2	5'-GGGGACCACTTTGTACAAGAAAGCTGGGTCCGCCATGGTGGAAGTGAAGTACGAATG-3'
LDH2 -1509 attB1	5'-GGGGACAAGTTTGTACAAAAAAGCAGGCTTCCCCGTAAATGTTCTCTACTTCCCC-3'
SAG 4.2 attB2	5'-GGGGACCACTTTGTACAAGAAAGCTGGGTCTGCCATTTTGCACACCGCGCGTCGTTG-3'
SAG 4.2 -1609 attB1	5'-GGGGACAAGTTTGTACAAAAAAGCAGGCTTCCACGGAATACCGCTGCGTCAGTCAG-3'
SAG4A attB2	5'-GGGGACCACTTTGTACAAGAAAGCTGGGTCCGCCATGGTTGAAGACAGACGAAAGCAG-3'
SAG4A -1500 attB1	5'-GGGGACAAGTTTGTACAAAAAAGCAGGCTTCGTCTGCTTGAACGGCGCACAAAGGC-3'
BAG1 attB2	5'-GGGGACCACTTTGTACAAGAAAGCTGGGTCCGCCATCTTTTTTGAATATCATACG--3'
BAG1 -1195 attB1	5'-GGGGACAAGTTTGTACAAAAAAGCAGGCTTCCTACCTTCTTCTCGATGTAGC-3'
BRS4 attB2	5'-GGGGACCACTTTGTACAAGAAAGCTGGGTCCGCCATTTTGTCTGACGCGCGTGCACCG-3'
BRS4 -1500 attB1	5'-GGGGACAAGTTTGTACAAAAAAGCAGGCTTCGCGCTTCCAAGTGTTCCCTCACGC-3'
B-NTPase attB2	5'-GGGGACCACTTTGTACAAGAAAGCTGGGTCCGCCATGATGGCGTATCATTC ACGACATGTAGTGTG-3'
B-NTPase -1495 attB1	5'-GGGGACAAGTTTGTACAAAAAAGCAGGCTTCCACTCGTGCTACAGTGTTGACACC-3'
Cyst Wall attB2	5'GGGGACCACTTTGTACAAGAAAGCTGGGTCCGCCATGTTGCTGTGCACAGTAACGCA-3'
Cyst Wall -1500 attB1	5'-GGGGACAAGTTTGTACAAAAAAGCAGGCTTCCTGGTTGTCGCCGCCTTCGGC-3'

Table S2 (Continued) . Primers used (unique to each figure).

Figure 4A Primers	
BAG1 -577 attB1	5'-GGGGACAAGTTTGTACAAAAAAGCAGGCTTCCAGAAGCTCTTCTTCGCCTTC-3'
BAG1 -524 attB1	5'-GGGGACAAGTTTGTACAAAAAAGCAGGCTTCCATCAGTCCTGAACTCCATG-3'
BAG1 -476 attB1	5'-GGGGACAAGTTTGTACAAAAAAGCAGGCTTCCGCAGCGAGTCGCGAGATTG-3'
BAG1 -457 attB1	5'-GGGGACAAGTTTGTACAAAAAAGCAGGCTTCGCCGGCCTGTCGACGAGTTCC-3'
BAG1 -441 attB1	5'-GGGGACAAGTTTGTACAAAAAAGCAGGCTTCGTTCCCGTTTTTCCTGCACTG-3'
BAG1 -428 attB1	5'-GGGGACAAGTTTGTACAAAAAAGCAGGCTTCCTGCACTGGCTACAGCGTAG-3'
BAG1 -408 attB1	5'-GGGGACAAGTTTGTACAAAAAAGCAGGCTTCCGGCTTCGCCTGCTCACTGGC-3'
BAG1 -385 attB1	5'-GGGGACAAGTTTGTACAAAAAAGCAGGCTTCCGGTACTGGCCGCACGGTTTC-3'
BAG1 -365 attB1	5'-GGGGACAAGTTTGTACAAAAAAGCAGGCTTCCACGATCCTGAGGTCGGCGAGAT-3'
BAG1 -283 attB1	5'-GGGGACAAGTTTGTACAAAAAAGCAGGCTTCGAAGGCCGGAGCGTTTTCTCAGA-3'

Figure 4B Primers	
BAG1 -500 rev w/ Avr-II	5'-TATTCCTAGGCCAAACATGGAGTTCAGGA-3'
BAG1 -450 for w/ Avr-II	5'-ATAACCTAGGTGTCGACGAGTTCCCGTT-3'
BAG1 -457 rev w/ Avr-II	5'-TATTCCTAGGCAATCTCGCGACTCGCTG-3'
BAG1 -428 for w/ Avr-II	5'-ATAACCTAGGCTGCACTGGCTACAGCGT-3'
BAG1 -365 for w/ Avr-II	5'-ATAACCTAGGCACGATCCTGAGGTCGGCGAGAT-3'
BAG1 -409 rev w/ Avr-II	5'-TATTCCTAGGCTACGCTGTAGCCAGTGCAG-3'
BAG1 -343 rev w/ Avr-II	5'-TATTCCTAGGATCTCGCCGACCTCAGGATCGTG-3'
BAG1 -283 for w/ Avr-II	5'-ATAACCTAGGGAAGGCCGGAGCGTTTTCTCAGA-3'

Figure 4C Primers	
B-NTPase -1202 attB1	5'-GGGGACAAGTTTGTACAAAAAAGCAGGCTTCCAGTCCGTGGTGCTTCTCCGATCATG-3'
B-NTPase -995 attB1	5'-GGGGACAAGTTTGTACAAAAAAGCAGGCTTCGGTCTTTTATCAGCCTTGTGCCGC-3'
B-NTPase -802 attB1	5'-GGGGACAAGTTTGTACAAAAAAGCAGGCTTCGAGTATATGCTAGACTCACCCCAG-3'
B-NTPase -611 attB1	5'-GGGGACAAGTTTGTACAAAAAAGCAGGCTTCGGCGACAGAGTCCTCTACAAC TAAGTATGG-3'
B-NTPase -403 attB1	5'-GGGGACAAGTTTGTACAAAAAAGCAGGCTTCCCAGTGAGGAACACGCGACGTAGG-3'
B-NTPase -184 attB1	5'-GGGGACAAGTTTGTACAAAAAAGCAGGCTTCGTGTGCCACCTCCCGTTGCATGCAAGG-3'

Table S2 (Continued) . Primers used (unique to each figure).

Figure 4D Primers	
B-NTPase -484-473 w/ Avr-II	5'-TATTCCTAGGCGCCACAGGTAGCAGAAACAAC-3'
B-NTPase -484-473 w/ Avr-II	5'-ATAACCTAGGCAGTATGACGGTGCGTGTGTG-3'
B-NTPase -477-468 w/ Avr-II	5'-TATTCCTAGGCCGAGTTCGCCACAGGTAGCAG-3'
B-NTPase -477-468 w/ Avr-II	5'-ATAACCTAGGTGACGGTGCGTGTGTGCGTGCAG-3'
B-NTPase -472-463 w/ Avr-II	5'-TATTCCTAGGAGAGGCCGAGTTCGCCACAGG-3'
B-NTPase -472-463 w/ Avr-II	5'-ATAACCTAGGGTGCGTGTGTGCGTGCAGCGC-3'
B-NTPase -467-458 w/ Avr-II	5'-TATTCCTAGGTACTGAGAGGCCGAGTTCGCCAC-3'
B-NTPase -467-458 w/ Avr-II	5'-ATAACCTAGGTGTGTGCGTGCAGCGCACTCTAC-3'
B-NTPase -462-453 w/ Avr-II	5'-TATTCCTAGGCGTCATACTGAGAGGCCGAGTTC-3'
B-NTPase -462-453 w/ Avr-II	5'-ATAACCTAGGGCGTGCAGCGCACTCTACATCTG-3'
B-NTPase -457-448 w/ Avr-II	5'-TATTCCTAGGCGCACCGTCATACTGAGAGGC-3'
B-NTPase -457-448 w/ Avr-II	5'-ATAACCTAGGCAGCGCACTCTACATCTGGTGG-3'
B-NTPase -452-443 w/ Avr-II	5'-TATTCCTAGGACACACGCACCGTCATACTGAGAG-3'
B-NTPase -452-443 w/ Avr-II	5'-ATAACCTAGGCACTCTACATCTGGTGGTGTTTG-3'
B-NTPase -447-438 w/ Avr-II	5'-TATTCCTAGGCACGCACACACGCACCGTCATAC-3'
B-NTPase -447-438 w/ Avr-II	5'-ATAACCTAGGTACATCTGGTGGTGTTTGCATG-3'
B-NTPase -442-433 w/ Avr-II	5'-TATTCCTAGGCGCTGCACGCACACACGCACC-3'
B-NTPase -442-433 w/ Avr-II	5'-ATAACCTAGGCTGGTGGTGTTTGCATGTGTTATCGC-3'

Figure 5 Primers	
B-NTPase -528-478 w/ Avr-II	5'-TATTCCTAGGGGAAGAACTACACAGAGCACAG-3'
B-NTPase -528-478 w/ Avr-II	5'-ATAACCTAGGCCTCTCAGTATGACGGTGCGTG-3'
B-NTPase -477-441 w/ Avr-II	5'-TATTCCTAGGCCGAGTTCGCCACAGGTAGCAG-3'
NTPase -477-441 w/ Avr-II	5'-ATAACCTAGGCTCTACATCTGGTGGTGTTTGC-3'
LDH2 -708 attB1	5'-GGGGACAAGTTTGTACAAAAAAGCAGGCTTCGCGTTTGCACCTCGTTCGTGAC-3'
LDH2 -340-277 w/ Avr-II	5'-TATTCCTAGGCTGGTTATTTGTACGCCTCAGCCCG-3'
LDH2 -340-277 w/ Avr-II	5'-ATAACCTAGGCCGGACGTGGTCGATGCCGGC-3'
LDH2 -426-377 w/ Avr-II	5'-TATTCCTAGGGCACCAGGTGACTGGAGAGTC-3'
LDH2 -426-377 w/ Avr-II	5'-ATAACCTAGGGTGACAAGCGTCGGGCTGAGGC-3'

Table S2 (Continued) . Primers used (unique to each figure).

**Figure 6 Primers**	
5'-989DHFR-P attB1	5'-GGGGACAAGTTTGTACAAAAAAGCAGGCTTCCTTCTAAATCCGGCGACAGGCTGGTC-3'
3'-oriDHFR-P attB2	5'-GGGGACCACTTTGTACAAGAAAGCTGGGTCCGCCATCTTCCCAGAC ACGACAACGCCCCG-3'
DHFR-P Avr-II Bgl-II	5'-GAAGATCTTCCTAGGAAGAGAATGGAACTTAATGTCCACGTAGTTCGCGC-3'
DHFR-P Bgl-II	5'-GAAGATCTTTTCGACAGCACGAAACCTTGCATTCAAACCCGC-3'
1X B-NTPase -462-438 Bgl-II	5'-GATCTGTGCGTGTGTGCGTGCAGCGCACTCC-3'
1X B-NTPase -462-438 Avr-II	5'-CTAGGGAGTGCGCTGCACGCACACACGCACA-3'
2X B-NTPase -462-438 Bgl-II	5'-GATCTGTGCGTGTGTGCGTGCAGCGCACTCGTGCGTGTGTGCGTGCAGCGCACTCC-3'
2X B-NTPase -462-438 Avr-II	5'-CTAGGGAGTGCGCTGCACGCACACACGCACGAGTGCGCTGCACGCACACACGCACA-3'
3X B-NTPase -462-438 Bgl-II	5'-GATCTGTGCGTGTGTGCGTGCAGCGCACTCGTGCGTGTGTGCGTGCAGCGCACTCGTG CGTGTGTGCGTGCAGCGCACTCC-3'
3X B-NTPase -462-438 Avr-II	5'-CTAGGGAGTGCGCTGCACGCACACACGCACGAGTGCGCTGCACGCACACACGCACG AGTGCGCTGCACGCACACACGCACA-3'
1X BAG1 -386-355 Bgl-II	5'-GATCTCGGTACTGGCCGCACGGTTTCACGATCCTGAC-3'
1X BAG1 -386-355 Avr-II	5'-CTAGGTCAGGATCGTGAAACCGTGCGGCCAGTACCGA-3'
2X BAG1 -386-355 Bgl-II	5'-GATCTCGGTACTGGCCGCACGGTTTCACGATCCTGACGGTACTGGCCGCAC GGTTTCACGATCCTGAC-3'
2X BAG1 -386-355 Avr-II	5'-CTAGGTCAGGATCGTGAAACCGTGCGGCCAGTACCGTCAGGATCGTGAAA CCGTGCGGCCAGTACCGA-3'

**Table 1 Primers**	
B-NTPase mut 1	5'-CTGGCGATAACACATGCAAACACCACCAGATGTAGAGTGCGCTGCACG CACACACGTTTTGTCATACTGAGAGGCCGAGTTCGCC-3'
B-NTPase mut 2	5'-CTGGCGATAACACATGCAAACACCACCAGATGTAGAGTGCGCTGCACG CACATTTTCACCGTCATACTGAGAGGCCGAGTTC-3'
B-NTPase mut 3	5'-CTGGCGATAACACATGCAAACACCACCAGATGTAGAGTGCGCTGCACG TTTTCACGCACCGTCATACTGAGAGGCCG-3'
B-NTPase mut 4	5'-CTGGCGATAACACATGCAAACACCACCAGATGTAGAGTGCGCTGTTTTC ACACACGCACCGTCATACTGAGAG-3'
B-NTPase mut 5	5'-CTGGCGATAACACATGCAAACACCACCAGATGTAGAGTGCTTTTCACGC ACACACGCACCGTCATACTG-3'
B-NTPase mut 6	5'-CTGGCGATAACACATGCAAACACCACCAGATGTAGATTTTGCTGCACGC ACACACGCACCGTCATAC-3'
B-NTPase mut 7	5'-CTGGCGATAACACATGCAAACACCACCAGATGTATTGTGCGCTGCACG CACACACGCACCG-3'
BAG1 wo mut	5'-TATTCCTAGGAAACCGTGCGGCCAGTACCGTCGCCAGTGAGCAGGCGAAGC-3'
BAG1 mut1	5'-TATTCCTAGGAAACCGTGCGGCCAGTATTATCGCCAGTGAGCAGGCGAAGC-3'
BAG1 mut2	5'-TATTCCTAGGAAACCGTGCGGCCAACGCCGTCGCCAGTGAGCAGGCGAAGC-3'
BAG1 mut3	5'-TATTCCTAGGAAACCGTGCGGTTGGTACCGTCGCCAGTGAGCAGGCGAAGC-3'
BAG1 mut4	5'-TATTCCTAGGAAACCGTGTAACCAGTACCGTCGCCAGTGAGCAGGCGAAGC-3'
BAG1 mut5	5'-TATTCCTAGGAAACCACACGGCCAGTACCGTCGCCAGTGAGCAGGCGAAGC-3'
BAG1 mut6	5'-TATTCCTAGGAAGTTGTGCGGCCAGTACCGTCGCCAGTGAGCAGGCGAAGC-3'
BAG1 mut7	5'-TATTCCTAGGGGACCGTGCGGCCAGTACCGTCGCCAGTGAGCAGGCGAAGC-3'

Table S2 (Continued) . Primers used (unique to each figure).

Figure 7 Primers	
B-NTPase 467-433 WT	5'-TGACGGTGCGTGTGTGCGTGCAGCGCACTCTACAT-3'
B-NTPase 467-433 WT	5'-ATGTAGAGTGCGCTGCACGCACACACGCACCGTCA-3'
B-NTPase 467-433 mut	5'-TGACGGTGCGTGAAAACGTGCAGCGCACTCTACAT-3'
B-NTPase 467-433 mut	5'-ATGTAGAGTGCGCTGCACGTTTTCACGCACCGTCA-3'

Figure 8 Primers	
BAG1 -385-365 WT	5'-CACTGGCGACGGTACTGGCCGCACGGTTTCACG-3'
BAG1 -385-365 WT	5'-CGTGAAACCGTGCGGCCAGTACCGTCGCCAGTG-3'
BAG1 -385-365 mut	5'-CACTGGCGACGGAAAAAAAAACACGGTTTCACG-3'
BAG1 -385-365 mut	5'-CGTGAAACCGTGTTTTTTTTTCCGTCGCCAGTG-3'

Figure S2 Primers	
SAG1 -1502 attB1	5'-GGGGACAAGTTTGTACAAAAAAGCAGGCTTCCATTAAACGATCCGGGACGAC-3'
SAG1 attB2	5'-GGGGACCACTTTGTACAAGAAAGCTGGGTCCGCCATACAACCGTGTGTTTACACGAC-3'
Fruc 1,6 -1502 attB1	5'-GGGGACAAGTTTGTACAAAAAAGCAGGCTTCCCCGTCCCCTATGCGATTGAA-3'
Fruc 1,6 attB2	5'-GGGGACCACTTTGTACAAGAAAGCTGGGTCCGCCATGTGTAGCTTTTTTTATTGTCTG-3'
GRA6 -1500 attB1	5'-GGGGACAAGTTTGTACAAAAAAGCAGGCTTCCGTTGGAGACGAGATGGGTTG-3'
GRA6 attB2	5'-GGGGACCACTTTGTACAAGAAAGCTGGGTCCGCCATTTCGCCGACACTCCCAAGAAA-3'
ROP4 -1502 attB1	5'-GGGGACAAGTTTGTACAAAAAAGCAGGCTTCGCCATTCACGTATGAATTTGA-3'
ROP4 attB2	5'-GGGGACCACTTTGTACAAGAAAGCTGGGTCCGCCATGTTGGGAGGACTCACAAACAA-3'
GAPDH -1500 attB1	5'-GGGGACAAGTTTGTACAAAAAAGCAGGCTTCCTCGACTGCTGACAGGAAGAA-3'
GAPDH 83.m0003 attB2	5'-GGGGACCACTTTGTACAAGAAAGCTGGGTCCGCCATCTTGGCAAAGTGTCTGAC-3'
MIC3 -1500 attB1	5'-GGGGACAAGTTTGTACAAAAAAGCAGGCTTCTGTCCCTTAGGTGACACCCGC-3'
MIC3 attB2	5'-GGGGACCACTTTGTACAAGAAAGCTGGGTCCGCCATTTTTACCAGTGTTGGACA-3'
IMC1 -1500 attB1	5'-GGGGACAAGTTTGTACAAAAAAGCAGGCTTCGGAACAACTTTGTCTAAACAA-3'
IMC1 attB2	5'-GGGGACCACTTTGTACAAGAAAGCTGGGTCCGCCATTCTGACAACAAACAGGAAGAC-3'

Figure S3A Primers	
LDH2 -1302 attB1	5'-GGGGACAAGTTTGTACAAAAAAGCAGGCTTCGGTTCCATCGTACCCTGAATGG-3'
LDH2 -1107 attB1	5'-GGGGACAAGTTTGTACAAAAAAGCAGGCTTCGTGGAGGTGATGTTCAGGCAG-3'
LDH2 -925 attB1	5'-GGGGACAAGTTTGTACAAAAAAGCAGGCTTCGCTACACGAGCTTTTGTTGCAATGG-3'
LDH2 -708 attB1	5'-GGGGACAAGTTTGTACAAAAAAGCAGGCTTCGCGTTTGCACCTCGTTCGTGAC-3'
LDH2 -507 attB1	5'-GGGGACAAGTTTGTACAAAAAAGCAGGCTTCCGAAGTGTGCACGCTTTGCAAG-3'
LDH2 -291 attB1	5'-GGGGACAAGTTTGTACAAAAAAGCAGGCTTCGACGCGATATCTGTACCGGAC-3'

Table S2 (Continued) . Primers used (unique to each figure).

Figure S3B Primers	
LDH2 -509-472 w/ Avr-II	5'-TATTCCTAGGCGCGGTTGTAGAGAGAAGAACG-3'
LDH2 -509-472 w/ Avr-II	5'-ATAACCTAGGCATCATCACTGGCTGATGATGGTG-3'
LDH2 -482-472 w/ Avr-II	5'-TATTCCTAGGCTCCTTGCAAAGCGTGCACACTTCG-3'
LDH2 482-472 w/ Avr-II	5'-ATAACCTAGGGCATCATCACTGGCTGATGATGGTG-3'
LDH2 -471-427 w/ Avr-II	5'-TATTCCTAGGCAGCTAAGCTAGCTCCTTGCAAAG-3'
LDH2 -471-427 w/ Avr-II	5'-ATAACCTAGGGGGAGGTACTGCCCGGAGCATG-3'
LDH2 -424-414 w/ Avr-II	5'-TATTCCTAGGCCGCACCAGGTGACTGGAGAGTC-3'
LDH2 -424-414 w/ Avr-II	5'-ATAACCTAGGCGGAGCATGCGTCCGGTTCCAG-3'
LDH2 -403-393 w/ Avr-II	5'-TATTCCTAGGGCATGCTCCGGGCAGTACCTCC-3'
LDH2 -403-393 w/ Avr-II	5'-ATAACCTAGGGAACCAGCGCCGGCCGGTGACAAG-3'
LDH2 -391-381 w/ Avr-II	5'-TATTCCTAGGCTGGAACCGGACGCATGCTCCG-3'
LDH2 -391-381 w/ Avr-II	5'-ATAACCTAGGGCCGGTGACAAGCGTCGGGCTGAG-3'
LDH2 -376-326 w/ Avr-II	5'-TATTCCTAGGCGGCCGGCGCTGGTTCTGGAACC-3'
LDH2 -376-326 w/ Avr-II	5'-ATAACCTAGGGCTCTGCAGGGGAGTGAAACTG-3'
LDH2 -340-277 w/ Avr-II	5'-TATTCCTAGGCTGGTTATTTGTACGCCTCAGCCCG-3'
LDH2 -340-277 w/ Avr-II	5'-ATAACCTAGGCCGGACGTGGTCGATGCCGGC-3'

Figure S3C Primers	
SAG 4.2 -1400 attB1	5'-GGGGACAAGTTTGTACAAAAAAGCAGGCTTCGGACTTTCGGTGTGGCACTTGAC-3'
SAG 4.2 -1207 attB1	5'-GGGGACAAGTTTGTACAAAAAAGCAGGCTTCGGCTGAACGGCGTACGAAAGG-3'
SAG 4.2 -973 attB1	5'-GGGGACAAGTTTGTACAAAAAAGCAGGCTTCGGCCACTCCCTCCGTGATATACAG-3'
SAG 4.2 -784 attB1	5'-GGGGACAAGTTTGTACAAAAAAGCAGGCTTCGGTTCTGAGACATTCCGATCCTTG-3'
SAG 4.2 -616 attB1	5'-GGGGACAAGTTTGTACAAAAAAGCAGGCTTCGGTCGTACAATGTTGTGTACGG-3'
SAG 4.2 -416 attB1	5'-GGGGACAAGTTTGTACAAAAAAGCAGGCTTCGCGTGTCAAGACCTAGACAGC-3'
SAG 4.2 -244 attB1	5'-GGGGACAAGTTTGTACAAAAAAGCAGGCTTCCTGACGCTTCGCCCGTTTCTC-3'

Figure S3D Primers	
Cyst Wall –1501 attB1	5'-GGGGACAAGTTTGTACAAAAAAGCAGGCTTCCTGGTTGTCGCCGCCTTCGGC-3'
Cyst Wall –1300 attB1	5'-GGGGACAAGTTTGTACAAAAAAGCAGGCTTCCGAAACAAGCTGACACTCACT-3'
Cyst Wall –1099 attB1	5'-GGGGACAAGTTTGTACAAAAAAGCAGGCTTCGAGACGAGGGCAAACAACCTG-3'
Cyst Wall –889 attB1	5'-GGGGACAAGTTTGTACAAAAAAGCAGGCTTCGGAACGACGCTCATCAGCAATC-3'
Cyst Wall –688 attB1	5'-GGGGACAAGTTTGTACAAAAAAGCAGGCTTCCGCTTACAGAGAGCCTGCAAG-3'
Cyst Wall –491 attB1	5'-GGGGACAAGTTTGTACAAAAAAGCAGGCTTCCCGACTGGCACTGTGTATATC-3'
Cyst Wall –265 attB1	5'-GGGGACAAGTTTGTACAAAAAAGCAGGCTTCCGAAGATGATGGCACTCGGTC-3'
